# Unexpected Ancient Paralogs and an Evolutionary Model for the COPII Coat Complex

**DOI:** 10.1093/gbe/evv045

**Published:** 2015-03-05

**Authors:** Alexander Schlacht, Joel B. Dacks

**Affiliations:** Department of Cell Biology, University of Alberta, Edmonton, Alberta, Canada

**Keywords:** protist, vesicle transport, protocoatomer, Sec13, Sec24, *Arabidopsis*

## Abstract

The coat protein complex II (COPII) is responsible for the transport of protein cargoes from the Endoplasmic Reticulum (ER) to the Golgi apparatus. COPII has been functionally characterized extensively in vivo in humans and yeast. This complex shares components with the nuclear pore complex and the Seh1-Associated (SEA) complex, inextricably linking its evolution with that of the nuclear pore and other protocoatomer domain-containing complexes. Importantly, this is one of the last coat complexes to be examined from a comparative genomic and phylogenetic perspective. We use homology searching of eight components across 74 eukaryotic genomes, followed by phylogenetic analyses, to assess both the distribution of the COPII components across eukaryote diversity and to assess its evolutionary history. We report that Sec12, but not Sed4 was present in the Last Eukaryotic Common Ancestor along with Sec16, Sar1, Sec13, Sec31, Sec23, and Sec24. We identify a previously undetected paralog of Sec23 that, at least, predates the archaeplastid clade. We also describe three Sec24 paralogs likely present in the Last Eukaryotic Common Ancestor, including one newly detected that was anciently present but lost from both opisthokonts and excavates. Altogether, we report previously undescribed complexity of the COPII coat in the ancient eukaryotic ancestor and speculate on models for the evolution, not only of the complex, but its relationship to other protocoatomer-derived complexes.

## Introduction

The eukaryotic membrane trafficking system is a network of membrane-bound organelles and protein machinery for transporting material between them. This system is underpinned by the formation and fusion of coated vesicles that transport a variety of cargoes destined for various cellular compartments (e.g., Golgi, lysosomes, plasma membrane). The appearance of these compartments in the protoeukaryote would have marked a key step in the evolution of the eukaryotic cell, resulting in the compartmentalization of diverse biochemical processes and precise control over membrane dynamics between the organelles and with the plasma membrane (Cavalier-Smith 2002). Although cell biological and biochemical analyses have allowed us to derive a mechanistic understanding of these processes in model organisms, that is, yeast and humans, comparative genomic analysis has provided the ability to discern between those features that are the result of lineage-specific modification, and those that are necessary for functional transport in diverse eukaryotic organisms. For example, past analyses have shown that many of the major coat forming complexes (coat protein complex I [COPI], adaptins, retromer, and TSET) and much of the machinery involved in membrane traffic (SNAREs, multisubunit tethering complexes, GTPases, etc.) are present in representatives of the major eukaryotic lineages (Koumandou et al. 2007, 2011; Hirst et al. 2011; Elias et al. 2012; Gabernet-Castello et al. 2013), suggesting that they are necessary for the proper function of the trafficking system, and providing insight into the mechanism of nonendosymbiotic organelle evolution (Field et al. 2007; Dacks et al. 2008; Field and Dacks 2009). In this mechanistic model, dubbed the Organelle Paralogy Hypothesis (OPH), the combination of gene duplication and coevolution of proteins encoding organelle identify and trafficking specificity (e.g., vesicle coat proteins, Rabs, SNAREs, ArfGAPs, and others) allows for the evolution of complexity and specialization of endomembrane organelles (Dacks and Field 2007). These types of analyses also allow us to make inferences about how this system evolved, particularly about which components were present in the Last Eukaryotic Common Ancestor (LECA) and how its trafficking system may have functioned. It has become apparent that the LECA possessed many of the major membrane trafficking genes known from studies in animals and fungi, indicating that it had a highly complex trafficking system.

The coat protein complex II (COPII) is responsible for the exit of proteins and lipids synthesized in the ER and their subsequent transport to the Golgi (Barlowe et al. 1994). COPII is composed of seven subunits that are sequentially recruited to form a budding vesicle. First, an ER localized guanine nucleotide exchange factor (GEF), Sec12, activates the small GTPase Sar1, by exchanging GDP for GTP. Activated Sar1 then binds the ER membrane and recruits the heterodimeric Sec23/24 adaptor complex which constitute the Sar1-GTPase activating protein (GAP) and primary cargo binding subunit, respectively. Recruitment of the Sec23/24 complex results in the recruitment and binding of the heterotetrameric Sec13/31 cage complex, thought to be responsible for membrane deformation. Sec16 is a multifunctional protein, implicated in the negative regulation of Sar1, controlling the timing of GTP hydrolysis (Kung et al. 2012), and acts as a scaffold, aiding the recruitment of the other COPII subunits (Gimeno et al. 1995, 1996; Shaywitz et al. 1997). Like Sec12, Sec16 is also excluded from the budding vesicle. Once free from the ER membrane, Sar1 hydrolyzes GTP and is released from the vesicle; however, the remainder of the coat remains largely intact (Lord et al. 2011). Following release of Sar1, the multisubunit tethering complex TRAPPI is recruited to vesicle through interaction with Sec23 (Cai et al. 2007). Interaction with the long-distance coiled-coil tether p115/Uso1p brings the coated vesicle in range of Golgi membrane binding by TRAPPI. Subsequent phosphorylation of the COPII coat is thought to stimulate coat disassembly, freeing vesicle localized SNAREs to interact with SNAREs on the Golgi membrane, resulting in fusion and membrane mixing (Lord et al. 2011).

The COPII complex is part of a larger family of membrane deforming complexes, including the other coat complexes (COPI, AP1-5, TSET), the intraflagellar transport system (IFT), the SEA complex, the HOPS (homotypic fusion and vacuole protein sorting)/CORVET (class C core vacuole/endosome tethering) tethering complex, and the nuclear pore complex (NPC). These seemingly different gene families are linked through the “protocoatomer domain architecture,” a protein fold composed of a β-propeller followed by an α-solenoid, common to all of these complexes. This is the basis for the protocoatomer hypothesis (Devos et al. 2004; Field et al. 2011), which posits the existence of an ancient membrane-deformation complex, far predating the LECA, that gave rise to all of the complexes mentioned above, implicitly under the processes described by the OPH mechanism. Understanding the relationship between these complexes would help us to understand how a highly complex membrane trafficking system evolved from an ancestor with no internal membrane compartments.

Although the COPII complex has been studied in a variety of organisms, including *Pichia pastoris* (Payne et al. 2000)*, Trypanosoma brucei* (Demmel et al. 2011; Sealey-Cardona et al. 2014)*, Arabidopsis thaliana* (De Craene et al. 2014), humans (Iinuma et al. 2007), and *Saccharomyces cerevisiae* (Barlowe et al. 1994; Gimeno et al. 1996; Kung et al. 2012)*,* relatively little is known about this complex outside of the latter two. In an effort to expand the understanding of NPCs and coat forming complexes in a variety of protistan lineages, a previous comparative genomic analysis identified components of the COPII complex in a set of diverse eukaryotic taxa (Neumann et al. 2010). Their analysis found that Sar1, Sec13, Sec16, Sec23, Sec24, and Sec31 are found in all eukaryotes, and therefore were likely present in the LECA. This is consistent with previous large-scale analyses of the eukaryotic endomembrane machinery that found both Sar1 and Sec31 as highly conserved markers of the COPII coat across eukaryotic diversity (Dacks and Field 2004). We have extended these analyses by examining two additional COPII components (Sec12 and Sed4), expanding on the taxon sampling, including recently sequenced key taxa, and in contrast to previous efforts, using in-depth phylogenetic analysis to assess the evolution of each COPII component. We newly identify ancient paralogs of several COPII components, increasing the reconstructed complexity of the COPII complex that was likely present in the ancestor of eukaryotes. We derive an evolutionary model describing the progression of the COPII complex from its configuration in an early representative of the eukaryotic lineage to that reconstructed in the LECA. Finally, we propose a hypothesis for the relationships of the protocoatomer-derived complexes, delving back to dawn of the endomembrane system in eukaryotes.

## Materials and Methods

### Comparative Genomics

**Fig. 1.—:**
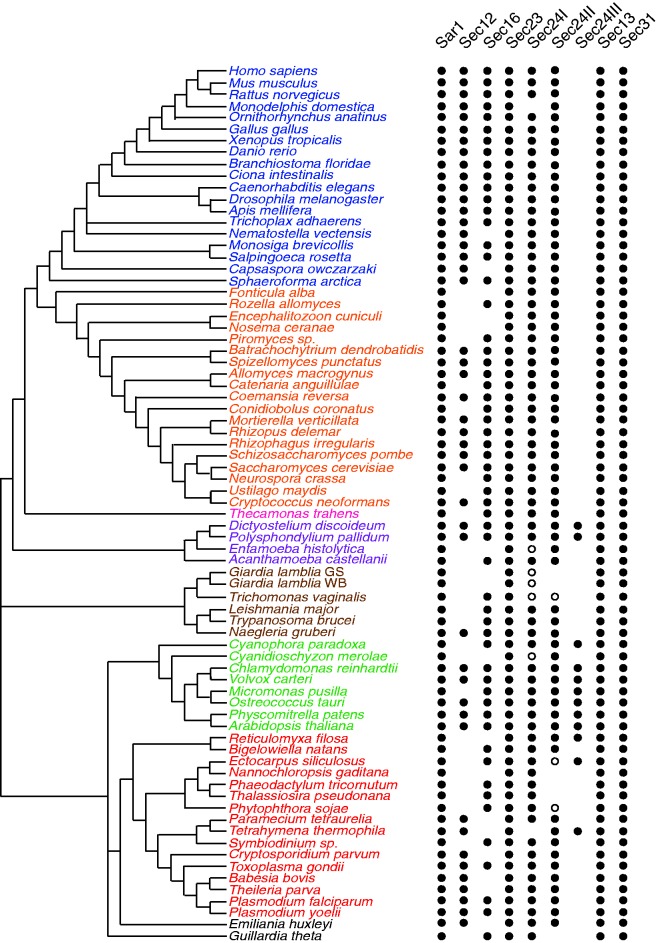
Comparative genomic analysis reveals the presence of COPII subunits across the diversity of eukaryotes. At least one ortholog of each Sar1, Sec23, Sec24, Sec13, and Sec31 has been identified in all taxa sampled, whereas Sec12 and Sec16 are missing from multiple eukaryotic taxa. All seven subunits are thought to have been present in the LECA. Black dots indicate the presence of at least one ortholog (column) in the corresponding organism (row), open dots represent additional Sec24 sequences that did not fall into any clade during phylogenetics and are classified based on best BLAST hit. Empty space indicates that no ortholog was identified. The broad distribution of all seven components suggests their presence in the LECA, with subsequent secondary loss of Sec12 and Sec16 in various lineages. Orthologous sequences were identified using BLAST and HMMer (see Materials and Methods). Relationships are based on molecular and ultrastructural data (Walker et al. 2011 inter alia).

COPII components from representative eukaryotic taxa were identified using Basic Local Alignment Search Tool (BLAST) (Altschul et al. 1997) against the proteomic databases of taxa in figure 1, with the *Homo sapiens* and *S. cerevisiae* sequences as queries. Orthology of candidate sequences was verified using the reciprocal best-hit method against both the *H. sapiens* and *S. cerevisiae* proteomic databases (Tatusov 1997; Bork et al. 1998). Sequences were considered orthologous if they retrieved either the *H. sapiens* or *S. cerevisiae* sequence as the top BLAST hit with an *E* value at least 2 orders of magnitude smaller than the next best hit.

In cases where BLAST failed to identify any orthologous sequences, Hidden Markov Models (HMMs) were generated for searching using HMMer v3.1b1 (http://www.hmmer.org, last accessed March 23, 2015). Multiple sequence alignments of orthologs identified using BLAST were generated using MUSCLE v3.6 (Edgar 2004). HMMs were built using the HMMbuild program from the HMMer suite and used to search proteomic databases using the HMMsearch program. Confirmation of orthology of the identified sequences was carried out as above.

In the event that HMMer also failed to identify orthologs for any of the COPII components, additional steps were taken to identify divergent sequences. First, organisms missing a given component were searched using the ortholog from the closest species with a positively identified sequence. Reciprocal confirmation of orthology was carried out as above, but with the reference genome being that of the query, rather than *H. sapiens* or *S. cerevisiae*. Second, if orthologs of any of the COPII components were still missing, the nucleotide databases (contigs/scaffolds) were searched using *H. sapiens*, *S. cerevisiae*, *Naegleria gruberi*, and *A. thaliana* queries and as reference genomes. Confirmation of orthology was carried out as above with reciprocal BLAST experiments carried out against each of the four genomes. For a list of sequences and accessions, see supplementary table S1, Supplementary Material online.

### Phylogenetics

Multiple sequence alignments were generated using MUSCLE and trimmed manually using Mesquite v2.75. Model testing was carried out using ProtTest v.3.3 (Guindon and Gascuel 2003; Darriba et al. 2011) and subsequent phylogenetic trees generated using PhyloBayes v1.5a (Lartillot et al. 2009) and RAxML v8.0.24 (Stamatakis 2006), bootstrapped with 100 pseudoreplicates. RAxML consensus trees were produced using the consense program from the Phylip package v3.695 (Felsenstein 2005). Phylogenetic trees were viewed using FigTree v1.4, and figures prepared using Adobe Illustrator CS4. For the size of alignments, evolutionary models, and substitution matrices used to generate each figure, see supplementary table S2, Supplementary Material online. All alignments are available upon request.

We used the Scrollsaw workflow (Elias et al. 2012) as implemented in Gabernet-Castello et al. (2013) to analyze the evolution of Sec23 and Sec24. All Sec23 and Sec24 sequences were combined into one data set and aligned using MUSCLE. The resulting alignment was trimmed manually using Mesquite. The trimmed data set was then broken into supergroup-specific data sets for phylogenetic analysis as above. From each supergroup data set, the two shortest branches from each clade were retained for a pan-eukaryotic analysis (clade defined as containing at least two sequences from at least two different organisms with minimal support from both methods; PhyloBayes = 0.8, RAxML = 50%). Sequences included in the pan-eukaryotic analysis were taken from the complete Sec23/24 alignment above, ensuring that the regions of the sequences used in each analysis were consistent. Phylogenetic analysis was carried out as above. All analyses were run on the CIPRES web server (Miller et al. 2010).

### Homology Modeling

Modeling of the *S. cerevisiae* Sed4 sequence was carried out using the Phyre v2.0 web server (Kelley and Sternberg 2009) on default settings. The structure was visualized using MacPyMOL (www.pymol.org, last accessed March 23, 2015).

## Results

### The Ancient Coat Complex COPII Has Both Sparsely and Ubiquitously Distributed Components

To assess the distribution of the COPII coat, we used BLAST and HMMer to identify orthologs of each component of the coat in a broad, representative distribution of eukaryotic genomes. Consistent with the results from Neumann et al. (2010), we identified at least one copy of Sar1, Sec23, Sec24, Sec13, and Sec31 in every eukaryotic genome analyzed (fig. 1), providing strong evidence that these subunits were present in the LECA. The pervasiveness of these proteins in diverse eukaryotic taxa highlights the key role that these five subunits play in forming the COPII coat, as seen from in vitro analyses, which have identified these five subunits as necessary and sufficient to bud vesicles from synthetic liposomes (Salama et al. 1993; Barlowe et al. 1994).

In contrast to the above subunits, we were unable to identify Sec12 and Sec16 in several taxa. Absences were not limited to one particular group of organisms, but were distributed across the five supergroups (fig. 1). Sec12 was missing more frequently than Sec16, and in only eight instances are they both missing in the same organism. Of these eight organisms, four (*Encephalitozoon cuniculi, Nosema ceranae, Entamoeba histolytica,* and *Giardia lamblia*) are parasites and are known for high levels of sequences divergence or cellular reduction, providing a possible explanation for the absence of these proteins. *Cyanidioschyzon merolae* is an extremophile with a minimal membrane trafficking system (Matsuzaki et al. 2004) and *Nannochloropsis gaditana* is a Eustigmatophycean microalga (Lubián 1982). These reduced cellular configurations likely resulted in a stripping down and loss of nonessential cellular machinery. In contrast, *Fonticula alba* and *Reticulomyxa filosa* are both free-living heterotrophs; therefore, sequence divergence is the most likely explanation for the absence of these two subunits, and may also be the case for the above genomes. In Sec16, only the central conserved domain is strongly conserved between taxa; therefore, sequence divergence in the flanking regions drastically increases the likelihood of false-negatives. This is also likely the case for Sec12; low sequence conservation and the presence of multiple WD40 repeats make it difficult to distinguish from other WD40 repeat containing proteins. This became apparent when trying to identify the *S. cerevisiae* Sec12 using the *H. sapiens* sequence; multiple rounds of psi-BLAST were required to show that they are indeed homologs, as BLASTp did not provide enough sensitivity to do so.

### Lineage-Specific Complement Expansions and an Ancient Sec23 Duplication

Phylogenetic analyses were carried out to determine the number paralogs of each COPII subunit present in the LECA, and to find expansions and reductions in various eukaryotic lineages (see Materials and Methods). Our analyses indicated that for five of the seven subunits analyzed, only one paralog was present in the LECA (supplementary figs. S1–S7, Supplementary Material online). These trees are characterized by one clade per supergroup, along with weak backbone support. As seen with other membrane-trafficking genes (Rutherford and Moore 2002; Sanderfoot 2007), all seven subunits have undergone expansions in multicellular plants (supplementary figs. S8–S14, Supplementary Material online) and five (Sar1, Sec23, Sec24, Sec31, and Sec16) have undergone expansions in vertebrates, correlating with increasing organismal complexity, possibly the result of selection for additional paralogs, permitting tissue specificity or differential regulation in these organisms.

We also observed an ancient duplication of Sec23. Our analysis uncovered two distinct Sec23 clades from the Archaeplastida, one of which is embedded in a group containing sequences from all other supergroups except the Excavata (supplementary fig. S15, Supplementary Material online). Although all of the sequences that make up this clade are long branches, possibly contributing to phylogenetic artifact, it is possible that this widely distributed, but sparsely encoded protein was present in the LECA. At a minimum, the two clades are the product of an ancient duplication that predates the archaeplastid lineage.

### Sed4 Is a Lineage-Specific Regulatory Component in a Subset of the Saccharomycotina

In addition to Sec12, *S. cerevisiae* possesses an additional Sec12-like protein, Sed4. Originally identified as a multicopy suppressor of Δ*erd2* (encodes HDEL receptor) (Hardwick et al. 1992), Sed4 is thought to aid in the recruitment of COPII components to the ER membrane by interacting with Sec16 (Gimeno et al. 1995) and act as a positive regulator of Sar1, likely by inhibiting the GTPase activity of Sec23 (Saito-Nakano and Nakano 2000). In contrast, other analyses suggest that Sed4 possesses GAP activity and is able to stimulate GTP hydrolysis on Sar1 when Bet1 is not bound to Sar1, suggesting a method for aborting COPII vesicles with low cargo density (Kodera et al. 2011). As *S. cerevisiae* is a major model organism for the study of COPII function, we wanted to address whether Sed4 is a general and ancient component of the complex.

**Fig. 2.—:**
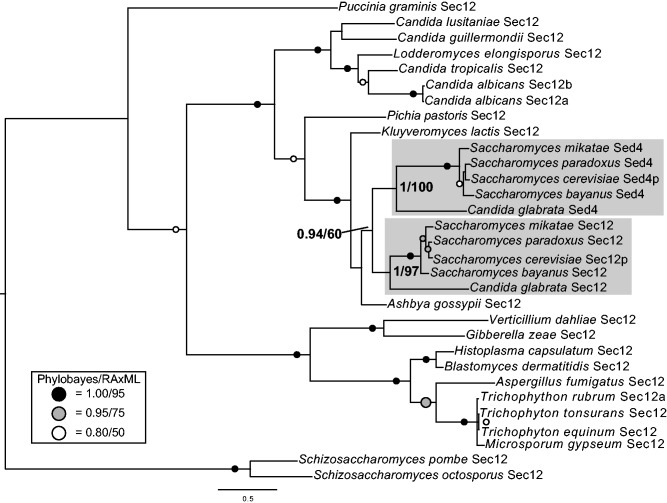
Phylogenetic analysis and homology modeling show that Sed4 is only found in a subset of the Saccharomycotina. Phylogenetic analysis of Sec12 and Sed4 sequences from representative fungal genomes shows that Sed4 is the product of a gene duplication in the ancestor of *C. glabrata* and *Saccharomyces* spp., not an ancient component of the COPII complex. Figure is the PhyloBayes topology. Values for critical nodes are shown (PhyloBayes posterior probabilities/RAxML maximum-likelihood), values for other nodes have been replaced by symbols, see inset.

In our initial survey, we did not find any Sed4 orthologs in any of our chosen taxa, indicating that the taxonomic distribution of this complex is limited compared with the other COPII subunits. In order to identify the origin and distribution of Sed4, we expanded our sampling of fungal taxa. BLAST results suggested that some fungi had either Sec12 or Sed4, and very few species seemingly possessed both. However, phylogenetic analysis revealed that most of these sequences are actually Sec12 orthologs, with Sed4 orthologs only in *Saccharomyces bayanus, Saccharomyces paradoxus, Saccharomyces mikatae*, and *Candida glabrata,* suggesting that the gene duplication generating Sed4 occurred in the ancestor of the *Saccharomyces* spp. and *C. glabrata* (fig. 2).

In order to gain some additional insight into the biology of Sed4, we decided to model its secondary structure. Homology modeling is generally reliable when the query and the primary sequence of a solved structure share at least 30% sequence identity (Xiang 2006; Kelley and Sternberg 2009). We predicted that, between the high level of sequence identity between Sed4 and Sec12 (45%) (Hardwick et al. 1992) and the recently solved structure of the cytosolic portion of Sec12 (McMahon et al. 2012), we could obtain a reliable structural prediction for Sed4. Use of the Phyre2 server (Kelley and Sternberg 2009) to model the structure of Sed4 identified the structure of Sec12 as the best homolog from which to model Sed4. Phyre was able to model 32% of Sed4 (corresponding to the cytosolic portion) with 100% confidence. The low coverage is due to the cytosolic portion being the region of homology between Sec12 and Sed4, whereas the extended luminal domain of Sed4 does not seem to share any sequence similarity to the luminal domain of Sec12, likely skewing the result. Although Sed4 has lost the ability to act as a Sar1 GEF (Saito-Nakano and Nakano 2000), it has retained structural similarity to Sec12, as suggested by homology modeling (supplementary fig. S16, Supplementary Material online). Sed4 is a beta-propeller protein, as has been proposed previously (Chardin and Callebaut 2002), and possesses a predicted K-loop, a short loop at the N-terminal propeller that binds a K^+^ thought to be important for the interaction of Sec12 with Sar1, suggesting that Sed4 may interact with Sar1 by a similar mechanism. In sum, rather than an ancient, widespread subunit, the evidence suggests that Sed4 is a recently added regulatory component to the COPII complexes in a subset of fungi.

### Multiple Paralogs of Sec24 Were Present in the LECA

The phylogenetic analysis described above of the Sec24 tree (supplementary fig. S3, Supplementary Material online) failed to show backbone resolution between paralogs. However, we did notice recurring clades (Holozoa, Fungi, SAR/CCTH, and Archaeplastida), suggesting that more than one paralog of Sec24 may have been present in the LECA. To test this hypothesis, we employed Scrollsaw, a phylogenetic approach that has been shown to produce backbone resolution between paralogs of large gene families where only short regions of homology are available (Elias et al. 2012; Gabernet-Castello et al. 2013). The extensive structural (Bi et al. 2002) and sequence similarity (Yoshihisa et al. 1993) between Sec23 and Sec24 suggest common ancestry (Tang et al. 1999). We therefore included the Sec23 sequences into this analysis to use as an outgroup for Sec24. We constructed an alignment of all Sec24 and Sec23 sequences, which was then broken into supergroup-specific data sets and analyzed (supplementary figs. S17–S21, Supplementary Material online; see Materials and Methods).

Previous analyses of Sec24 had suggested important duplication events in the history of this component (Pagano et al. 1999; Tang et al. 1999); alignments and phylogenetic analyses showed that the human Sec24A, Sec24B, and *S. cerevisiae* Sec24p are more similar and group separately from the human Sec24C, Sec24D, and *S. cerevisiae* Sfb2 and Sfb3 sequences. This suggested that there were likely at least two paralogs of Sec24 in opisthokonts, and that these groups of paralogs represent the descendants of those lineages. In each of our phylogenetic analyses (supplementary figs. S17–S21, Supplementary Material online), the Sec24 sequences were resolved into two major clades. Based on the reciprocal best hit against the human genome, these largely corresponded to those that preferentially retrieved *H. sapiens* Sec24A and B, and those that retrieved Sec24C and D. To differentiate the two clades, we have given them the names Sec24I, corresponding to the group containing *H. sapiens* Sec24A and B, and Sec24II, corresponding to the group containing *H. sapiens* Sec24 C and D.

Next, the two shortest branches from each clade, including Sec23, were retained for use in a pan-eukaryotic phylogenetic analysis (carried out as above), with the selected sequences acting as surrogates for the rest of the supergroup (supplementary fig. S22, Supplementary Material online). Most supergroups possessed two Sec24 clades along with other unclassified Sec24 sequences. Rooting with Sec23 resulted in a paraphyletic Sec24I cluster, which we suspected was a misplacement of the root. We therefore removed all Sec23 sequences from the analysis (supplementary fig. S23, Supplementary Material online), which clearly shows two distinct Sec24 clades.

Upon analyzing the supergroup-specific data set for the Archaeplastida, we noticed a third clade of Sec24 sequences that did not correspond to one of the paralogous sets found in Opisthokonta. We reexamined the other supergroup-specific data sets (supplementary figs. S17–S19 and S21, Supplementary Material online) to determine whether additional Sec24 clades were observed for the other supergroup-specific data sets. These were identified in data sets for both SAR and Amoebozoa (supplementary figs. S18 and S21, Supplementary Material online). Results from BLAST searches indicate that these paralogs retrieve each other as top BLAST hits rather than paralogs from Sec24I or Sec24II, suggesting that these sequences may represent another ancient paralog. To confirm that these sequences do not represent multiple convergent, lineage-specific expansions, we carried out a phylogenetic analysis of Sec24 sequences from only these taxa (supplementary fig. S24, Supplementary Material online), confirming that these sequences form a distinct lineage separate from the two known Sec24 paralogs, labeled Sec24III. Given the taxa present in this clade, we believe that this group represents yet another Sec24 paralog that was present in the LECA.

**Fig. 3.—:**
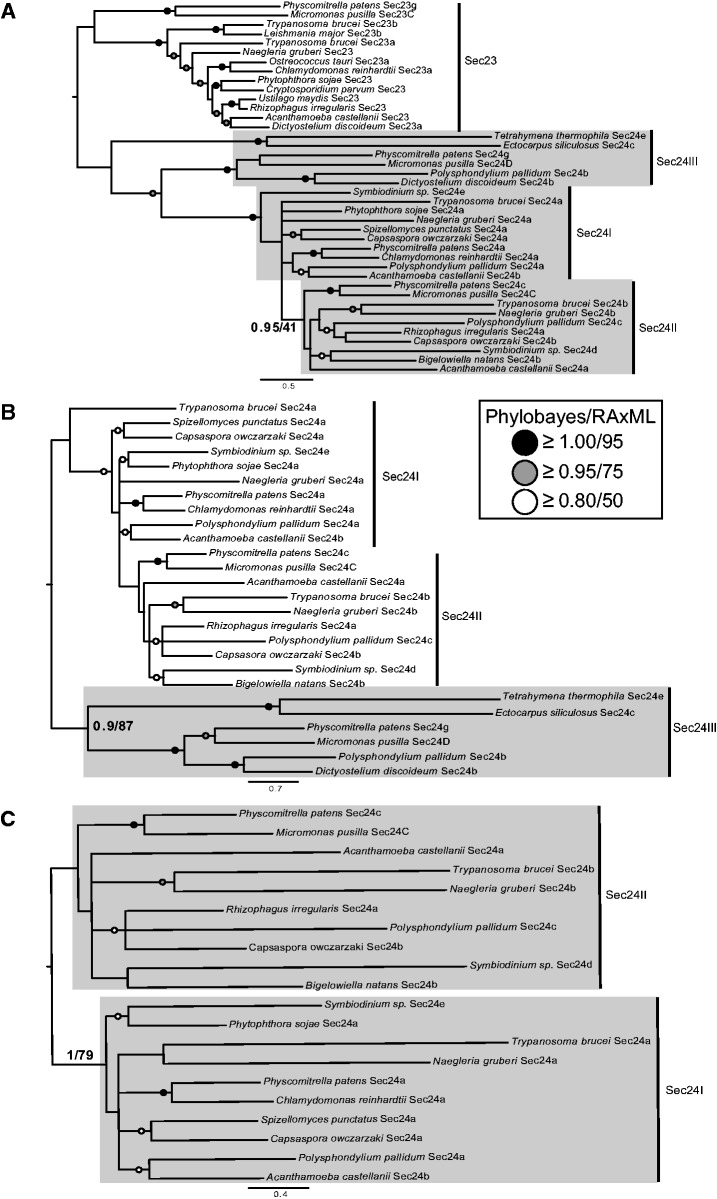
Scrollsaw analysis identifies three Sec24 paralogs in the LECA. (*A*) Final tree resulting from the Scrollsaw analysis, rooted on the Sec23 clade, shows two subclades of Sec23 and one weakly supported Sec24 clade (Sec24II). The other ancient Sec24 paralogs cluster together, but without support. (*B*) Scrollsaw tree with all Sec23 sequences removed and arbitrarily rooted on Sec24III for visualization purposes. Only the Sec24III clade is moderately supported to the exclusion of Sec24I and II. (*C*) Scrollsaw data set containing only Sec24I and Sec24II. Both clades are supported to the exclusion of the other. Trees are the PhyloBayes topology. Values for critical nodes are shown (PhyloBayes posterior probabilities/RAxML maximum-likelihood), values for other nodes have been replaced by symbols, see inset.

For each supergroup with Sec24III, we took the two shortest branches and added them into the final Scrollsaw data set (fig. 3*A*). Rooting on Sec23, we recovered a weakly supported Sec24II clade; however, we did not recover monophyletic Sec24I or Sec24III clades. Believing that the apparent lack of resolution is the result of long branch attraction coupled with large evolutionary distances, we removed all Sec23 sequences from the analysis (fig. 3*B*). In doing so, we recovered a moderately supported Sec24III clade, but no resolution between Sec24I and Sec24II. This was addressed by removing all Sec24III sequences, resulting supported Sec24I and Sec24II clades (fig. 3*C*). This analysis confirmed the presence of three Sec24 paralogs in the LECA. However, the lack of backbone support, and potential phylogenetic artifact in outgroup rooted analyses, prevented us from determining the order in which each paralog emerged.

## Discussion

Our comparative genomic analyses have shown that seven COPII subunits are broadly conserved across eukaryotes with Sar1, Sec23, Sec24, Sec13, and Sec31 found in all organisms analyzed, and Sec12 and Sec16 missing from multiple lineages. The broad distribution of these subunits suggests that this form of the COPII complex was present in LECA (Neumann et al. 2010). Our phylogenetic analyses have also allowed us to reconstruct the number of paralogs for each subunit present at that time. For five of the components, only one copy was present. Although the copy number of Sec23 as a single or double is equivocal, it is clear that three copies of Sec24 were present in the LECA, suggesting that Sec24 may have been one of the first drivers of complexity in this coat system. This is consistent with the function of Sec24 as the primary cargo binding subunit of the complex; multiple paralogs would have allowed for a greater diversity and specificity of cargo to be transported out by COPII. It has been observed that some Sec24 paralogs have multiple binding sites each with specificity for some sorting signals or cargoes (Miller et al. 2003; Mossessova et al. 2003; Wendeler et al. 2007; Sucic et al. 2011), suggesting that the cargo specificity of Sec24 paralogs evolved early on in the eukaryotic lineage. Multiple paralogs, each with multiple binding sites, would have drastically increased the number or specificity of cargo binding, and therefore many have been selected for. The LECA is thought to have been a biflagellated organism (Cavalier-Smith et al. 2014); however, it remains unclear what other lifecycle stages (i.e., amoeboid, etc.) it may have had. Should it be the case that the LECA underwent multiple lifecycle stages, encoding multiple differentially expressed forms of Sec24 would have added another layer of specificity enabling tighter regulation of the coat complex, as well as provide additional regulatory mechanisms for the various cargoes to be exported. The ancient Sec24III paralog is yet another example of ancient, patchy proteins that are found in diverse eukaryotic taxa, but that have been lost from opisthokonts (Elias et al. 2012; Gabernet-Castello et al. 2013; Schlacht et al. 2013, 2014; Hirst et al. 2014). The apparent asymmetry in the distribution of these proteins is suggestive of novel cell biology not found in typical model systems (mammals, yeast). As Sec24III is found in stramenopiles, plants, and amoebozoans, taxa with agricultural, and medical importance, the protein may represent a useful target for exploitation.

The observation that not only Sec16 but also Sec12 are widely distributed, but seemingly missing from some organisms, requires some reconciling with the functional data. Sec12 is a Sar1 GEF and is responsible for activating Sar1 by swapping GDP for GTP, recruiting it to the ER membrane. Sec16 on the other hand is a multifunctional scaffolding protein involved in both the recruitment of COPII subunits and has been implicated in regulating GTP hydrolysis (Kung et al. 2012). If these absences are in fact gene losses, then they have occurred multiple times independently, with Sec12 being lost much more frequently than Sec16. This is surprising as Sec12 essentially acts to initiate COPII coat formation, and that Sec16 has been shown to be essential for vesicle formation in vivo (Kaiser and Schekman 1990). Very few organisms are missing both subunits, and could suggest that lower levels of ER to Golgi trafficking are necessary in these organisms or that, given the appropriate cellular conditions, these factors are not necessary for the formation of COPII-coated vesicles. In this scenario, Sec12 and Sec16 would serve to increase the speed and efficiency of coat formation, rather than acting as integral steps in the process. Alternatively, other GEFs or scaffold proteins may have functionally replaced Sec12 or Sec16. Promiscuity between GAPs and their GTPases has previously been observed in vitro, with ELMOD2, an Arf-like protein GAP, being able to replace Arf GAPs and stimulate GTP hydrolysis on Arf (East et al. 2012). However, as mentioned above, it is likely that some of the reported failures to identify Sec12 and Sec16 represent cases of extreme divergence, and in vivo analyses may well identify divergent orthologs of these proteins.

**Fig. 4.—:**
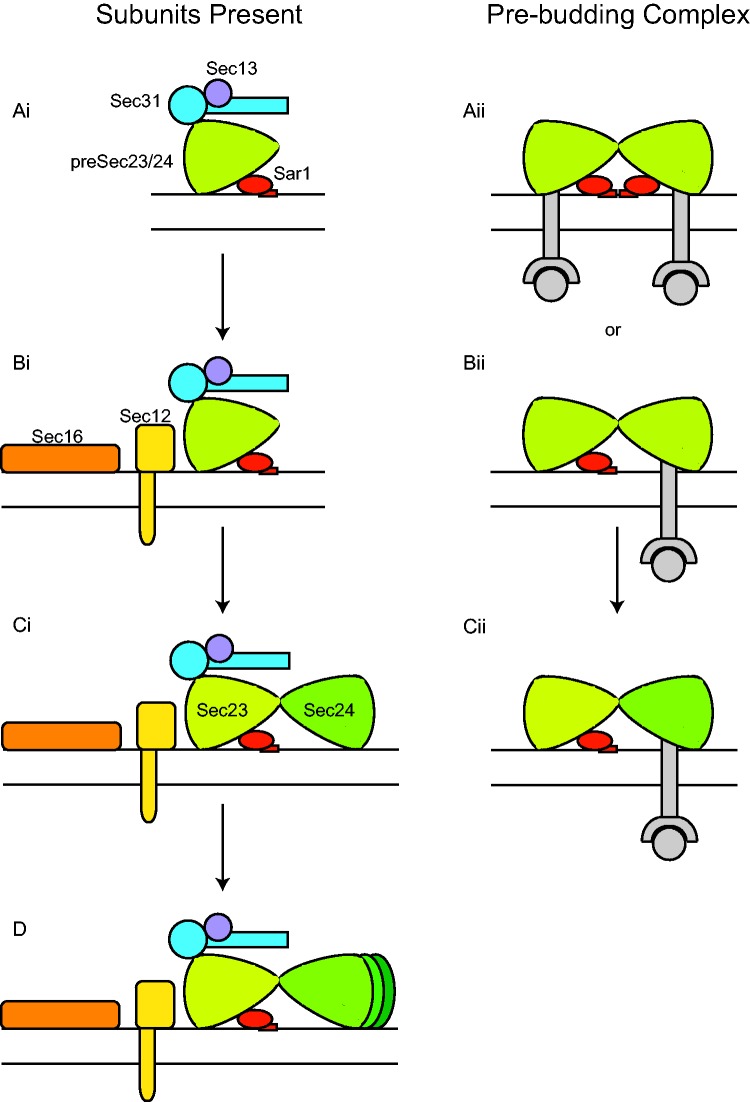
Proposed model for the evolution of the COPII complex from its earliest beginnings to the LECA. The left column represents the evolving complement of subunits present in the pre-eukaryotic lineage; the right column represents the evolution and hypothetical pre-budding complex across the same timeline. Note: this model is not meant to represent the stoichiometry or quaternary structure of individual subunits of the complex, but rather, is a hypothesis for the evolution of the complex itself. (Left column, subunits present) The earliest COPII coat was composed of Sar1, Sec13, Sec31, and a preduplicate of Sec23 and Sec24 (preSec23/24; Ai). Next, Sec12 and Sec16 would appear (Bi). Following this, a gene duplication of the preSec23/24 would have given rise to Sec23 and Sec24 (Ci). Finally, Sec24 would have undergone sequential gene duplications producing the three paralogues present in the LECA (D). (Right column, heteromeric complex) Two copies of preSec23/24 likely interacted during coat formation with two possibilities for protein binding: both copies of preSec23/24 may have been able to bind both Sar1 and cargo (Aii). Alternatively, one bound Sar1 and the other bound cargo (Bii). The precise configuration would have depended on the location of Sar and cargo binding sites present in the preSec23/24 subunit. From here, the duplication of preSec23/24 produced Sec23 and Sec24, resulting in the subfunctionalization and fixation of GAP activity and cargo binding into two distinct subunits (Cii).

Our phylogenetic analyses and reconstruction of the COPII coat in the LECA allows us to propose a model for the evolution of the coat from its state in early eukaryotes to its current incarnation in extant lineages; however, this model does not imply any precise stoichiometry or quaternary structure. The earliest form of COPII was likely made up of Sar1, Sec13, Sec31, and a preduplicate of Sec23 and Sec24 (preSec23/24; fig 4 Ai, Aii) that worked as a heteromeric complex. The preSec23/24 may have possessed both the Sar1-binding/GAP activity of Sec23 and the cargo binding capability of Sec24, suggesting that the preSec23/24 could bind both Sar1 and cargo. Alternatively, the preSec23/24 may have bound either Sar1 or cargo if these binding sites overlapped. Eventually, Sec12 and Sec16 would be added to the coat-forming process, increasing the speed and efficiency of vesicle formation (fig. 4Bi). Next, Sar1-binding/GAP activity and cargo binding were separated by the duplication of the preSec23/24 producing Sec23 and Sec24 (fig. 4Ci), possibly fixing their functions as the GAP and cargo binding subunits through subfunctionalization. Finally, iterative gene duplications would increase the cargo specificity and capacity of COPII by giving rise to the three paralogues of Sec24 present in the LECA (fig. 4*D*).

**Fig. 5.—:**
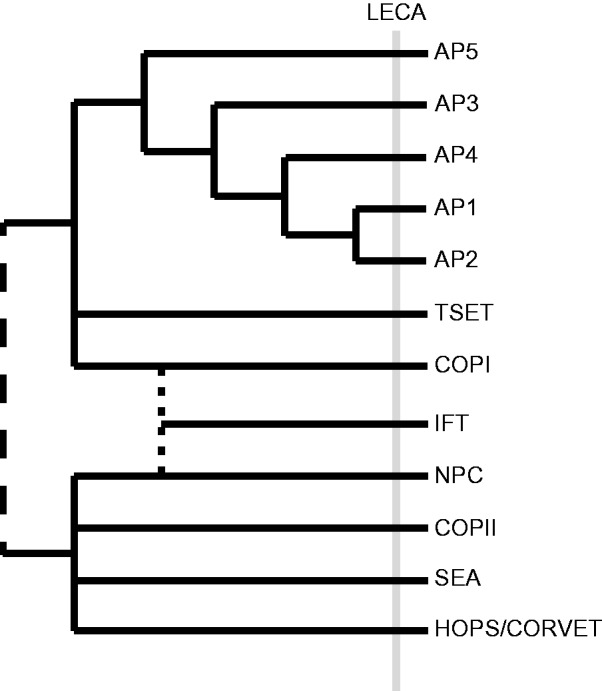
Proposed phylogenetic affinities of known protocoatomer domain-containing complexes. COPII, the NPC, the SEA complex, and HOPS/CORVET likely form a single group based on both the presence of Sec13 in multiple complexes and the presence of subunits that share similar structures to Sec31. COPI, TSET, and the APs form a distinct group based on their shared tetrameric structure. The dotted line denotes the uncertainty regarding the relationship of the intraflagellar transport complex to the other protocoatomer-derived complexes: A previous analysis suggests that it is sister to COPI,whereas others suggests that it may share a more recent common ancestor with the NPC (Kee and Verhey 2013; van Dam et al. 2013). The dashed line linking the upper clade to the other complexes emphasizes the uncertainty of this placement with respect to their interlatedness and the root of the protocoatomers.

An extended analysis of the COPII complex, together with recent discoveries of additional protocoatomer-related complexes gives us the opportunity to consider the possible relationships between the endomembrane organelles and the earliest steps in the evolution of the endomembrane system. It has been speculated that COPII and the NPC likely share more recent common ancestry than the other complexes due to their common component Sec13 (Field and Dacks 2009). The recently described SEA complex, with protocoatomer architecture proteins SEA2–4, also contains Sec13 and Seh1. As these are also NPC components, this additionally links the SEA complex and the other two (Dokudovskaya et al. 2011; Algret et al. 2014). SEA4 appears structurally most similar either to Sec31 or to Vps39 (Dokudovskaya et al. 2011), a component of the HOPS complex that also acts at the vacuole. Furthermore, the GEF for the GTPase that functions with the SEA complex (Gtr1) is Vps39. There is therefore a tentative evolutionary connection between HOPS to the SEA, COPII and NPC. Indeed, recent evidence increasingly supports both functional and evolutionary relationships between the organelles served by these complexes, the nuclear envelope/ER and vacuole, with proteins such as Rab32 (Sandoval and Simmen 2012), Syntaxin 17 (Itakura et al. 2012; Hamasaki et al. 2013), and PACS-2 (Simmen et al. 2005; Atkins et al. 2008) appearing to function in both locations. On the other hand, the newly described TSET complex is undoubtedly related to COPI and AP complexes (Hirst et al. 2014). Phylogenetic analyses, using concatenations of the subunit gene sequences, have been able to provide resolution of the appearance order of these vesicle coats, at least within the Adaptin heterotetramers with AP5, and then AP3 emerging basally, followed by AP4, then AP1 and 2. These various interconnections suggest a model with two groups in the protocoatomer “tree” (fig. 5). The relationship of the IFT complex to the other protocoatomers is somewhat more equivocal. Recent work links some IFT components to COPI (Van Dam et al. 2013). However, many microbial eukaryotes have a combined nuclear ciliary structure, the “karyomastigont” (Walker et al. 2011) inter alia, and in mammalian cells nucleoporins form a complex at the base of the cilium (Kee et al. 2012), all suggesting a functional and evolutionary connection between the IFT and the NPC (Kee and Verhey 2013). Testing all of these relationships phylogenetically and determining the placement of the root between them remain a difficult but important challenge, which has the potential to clarify much of the order of evolution of distinct structures in the pre-LECA cell.

Our analysis has allowed us to provide more general context to experimental characterization of the COPII coat, in model systems, to a wide range eukaryotes. Several previously undetected paralogs of COPII components have been identified in organisms of agricultural and medical relevance. Our analysis has also provided insight not only into the evolution of this coat complex in a variety of eukaryotic lineages, but also in the lineage leading up to the LECA. Although we do not yet know how the LECA’s membrane trafficking system was configured, the presence of the COPII complex suggests that exit from the ER occurred in a highly regulated manner, not unlike that observed in modern eukaryotes.

## Supplementary Material

Supplementary tables S1 and S2 and figures S1–S24 are available at *Genome Biology and Evolution* online (http://www.gbe.oxfordjournals.org/).

Supplementary Data
