# Supplementary material for: Unexpected Ancient Paralogs and an Evolutionary Model for the COPII Coat Complex
Source: Genome Biol Evol. 2015 Mar 5;7(4):1098–109. doi: 10.1093/gbe/evv045 (PMC4419792; doi:10.1093/gbe/evv045)
Supplement: Supplementary Data [file supp_7_4_1098__index.html]

Unexpected Ancient Paralogs and an Evolutionary Model for the COPII Coat Complex — Supplementary Data 

# Unexpected Ancient Paralogs and an Evolutionary Model for the COPII Coat Complex

## Supplementary Data

files

**Files in this Data Supplement:**

- Supplementary Data - pdf file
- Supplementary Data - xlsx file
- Supplementary Data - xlsx file
