# Supplementary material for: Unexpected Ancient Paralogs and an Evolutionary Model for the COPII Coat Complex
Source: Genome Biol Evol. 2015 Mar 5;7(4):1098–109. doi: 10.1093/gbe/evv045 (PMC4419792; doi:10.1093/gbe/evv045)
Supplement: Supplementary Data [file supp_evv045_Schlacht_Dacks_SupplFiguresS1-24.pdf]

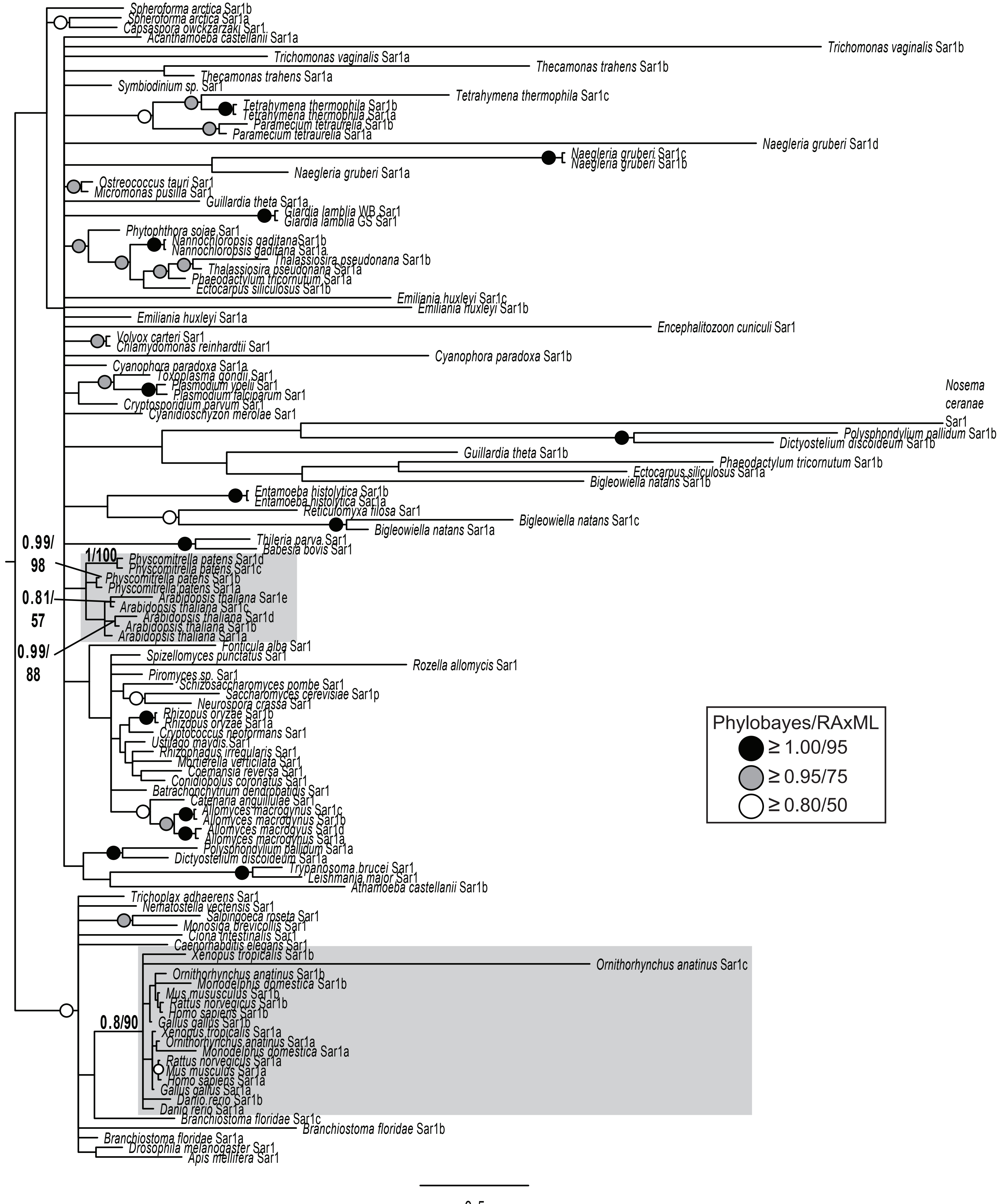

Schlacht and Dacks Figure S1

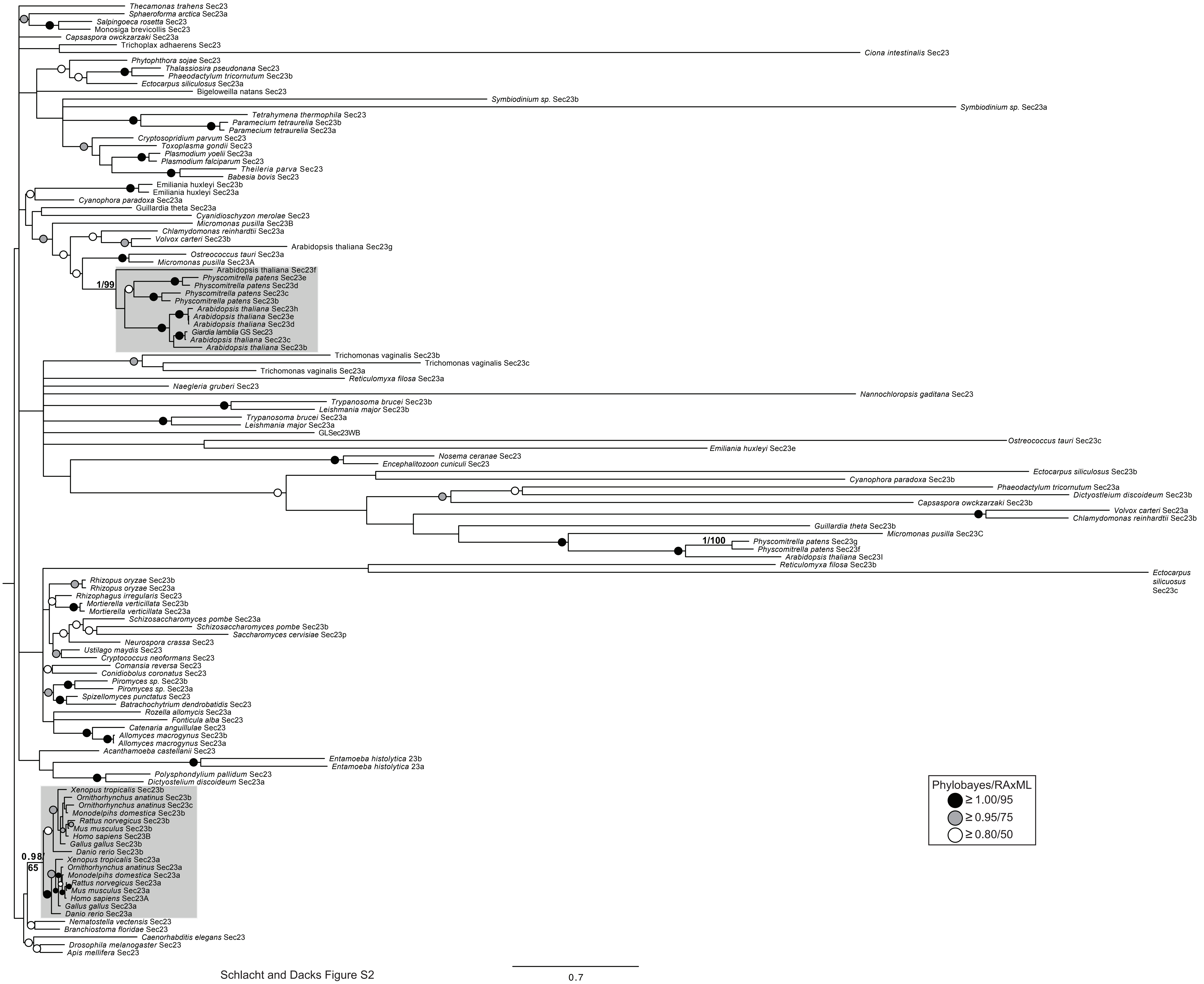

Schlacht and Dacks Figure S2

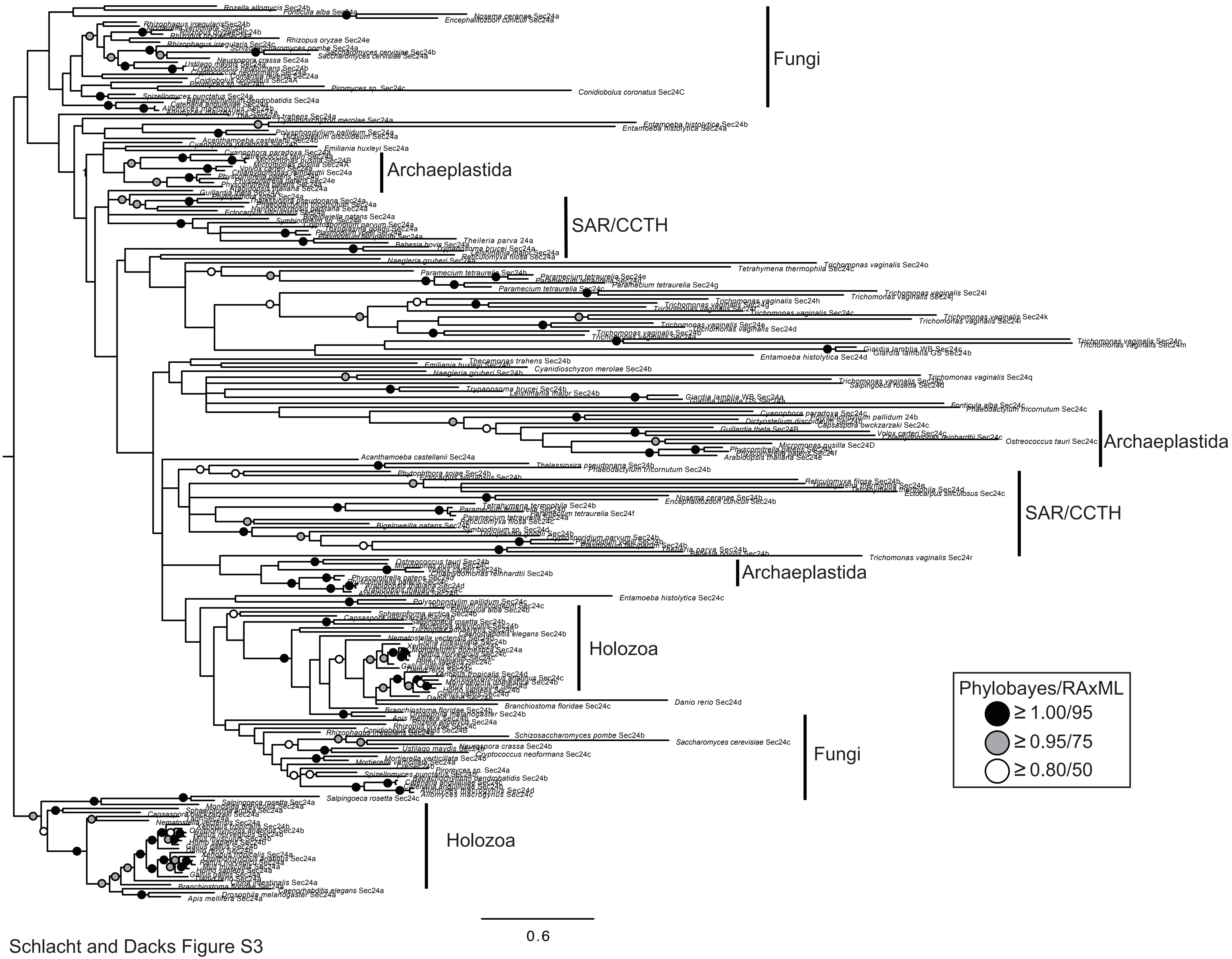

Schlacht and Dacks Figure S3

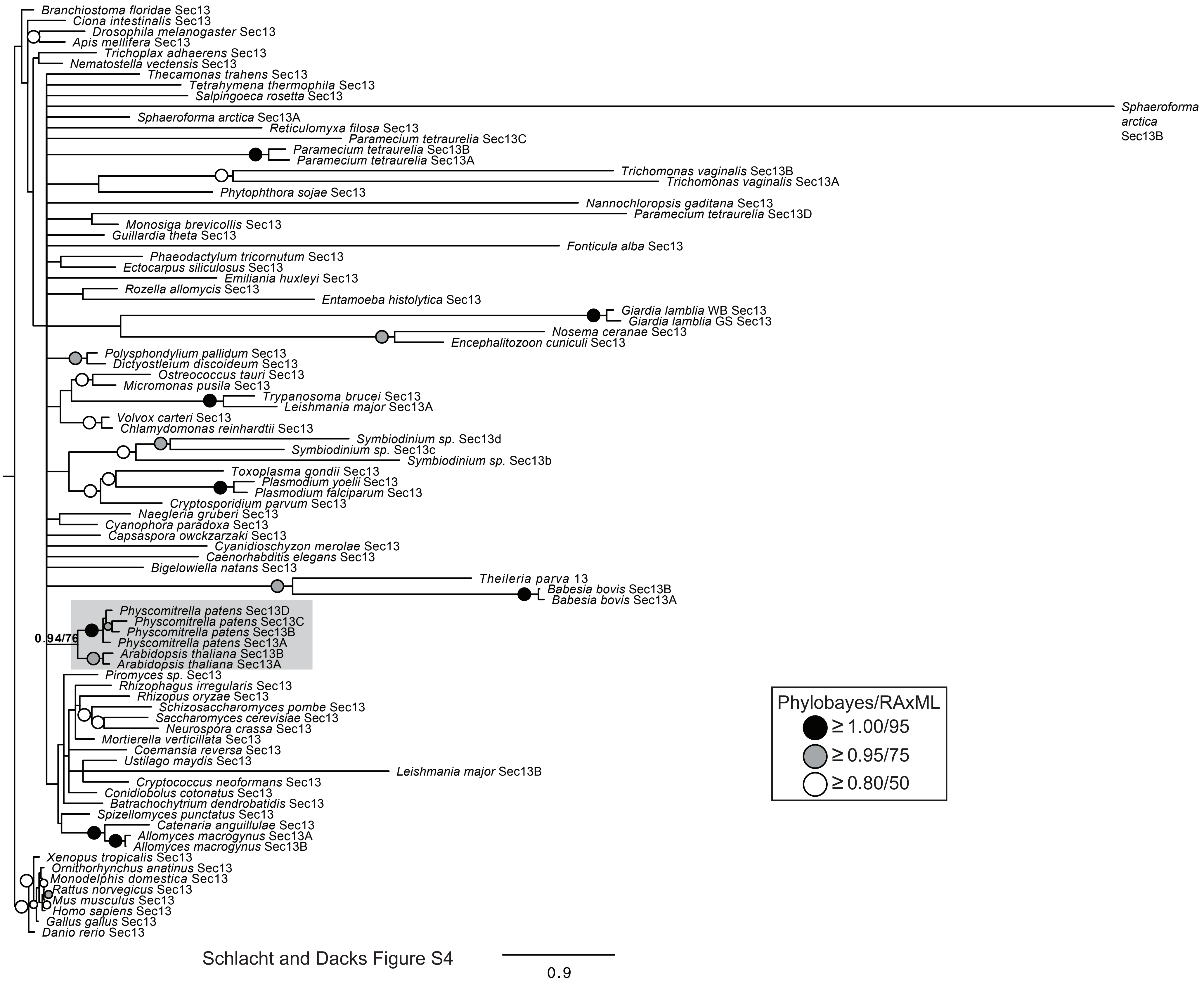

Schlacht and Dacks Figure S4

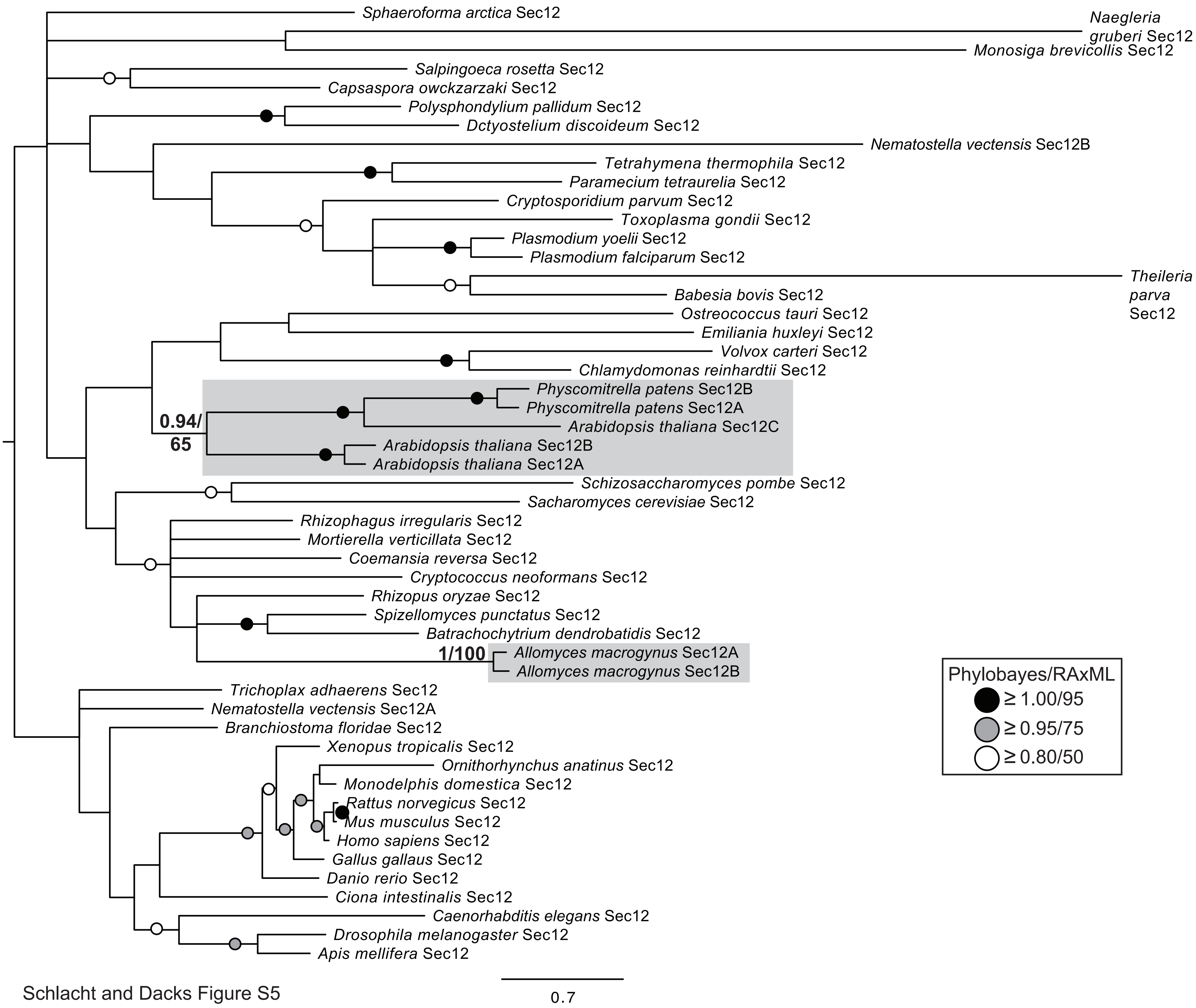

Schlacht and Dacks Figure S5

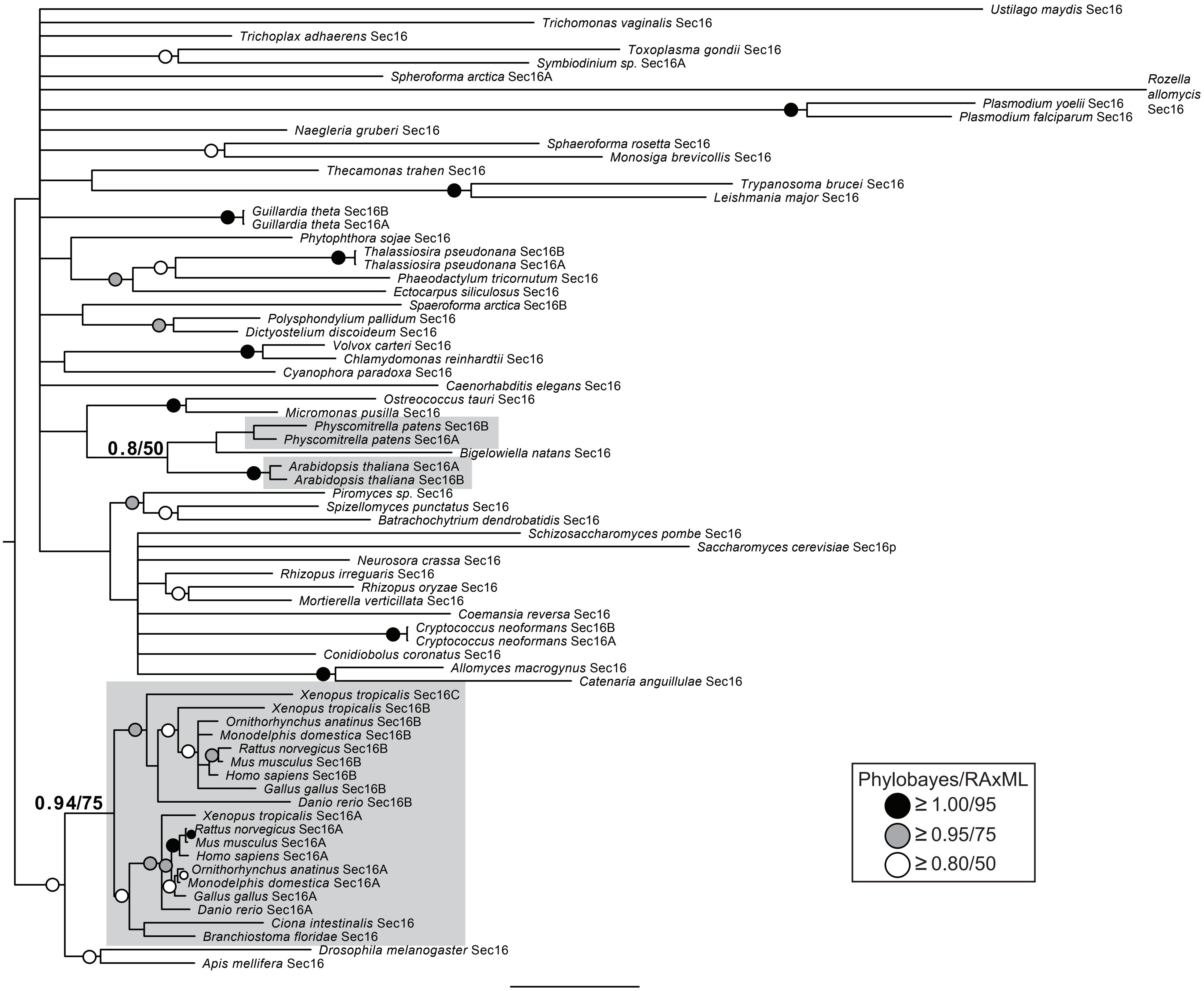

Schlacht and Dacks Figure S6

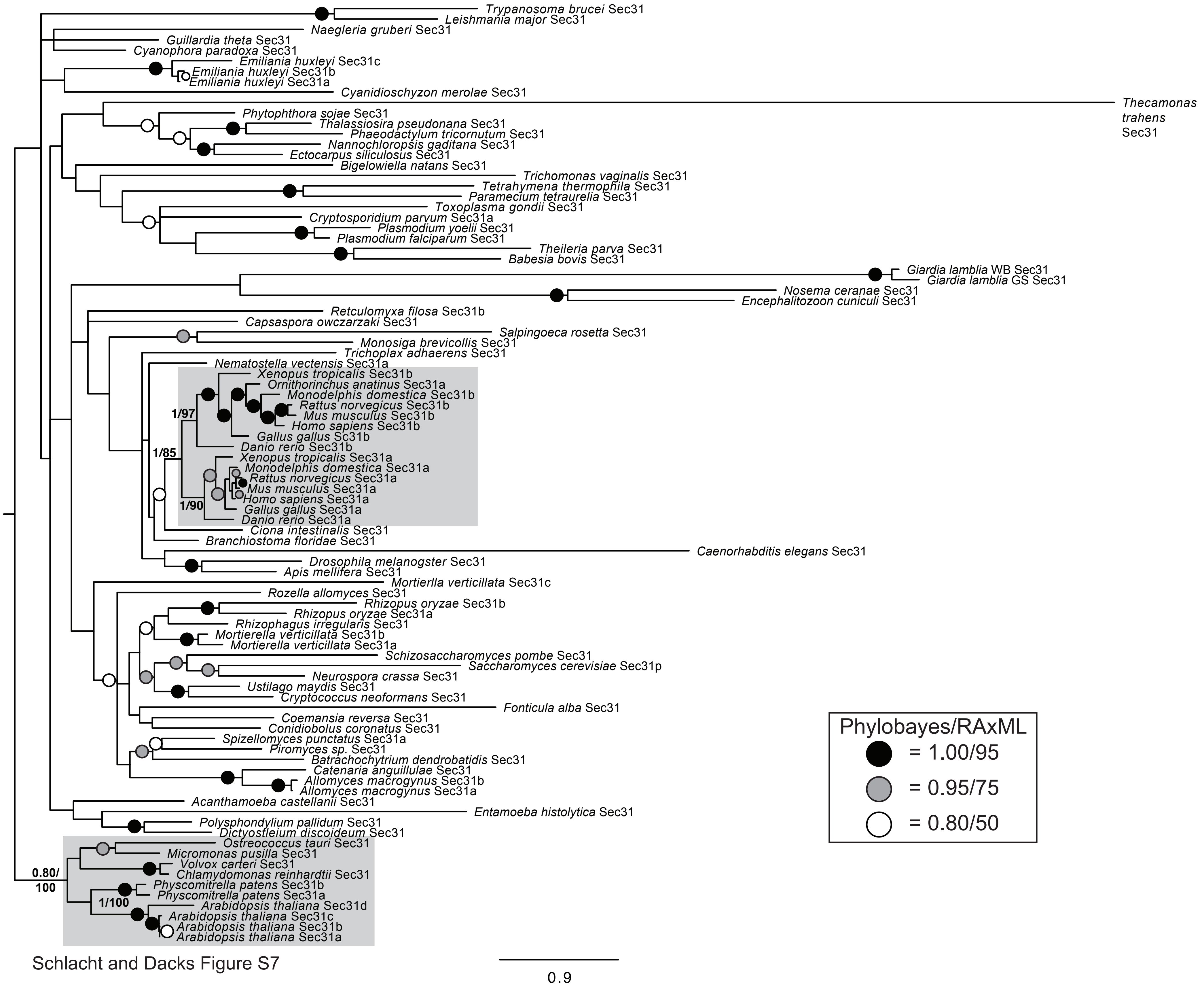

Schlacht and Dacks Figure S7

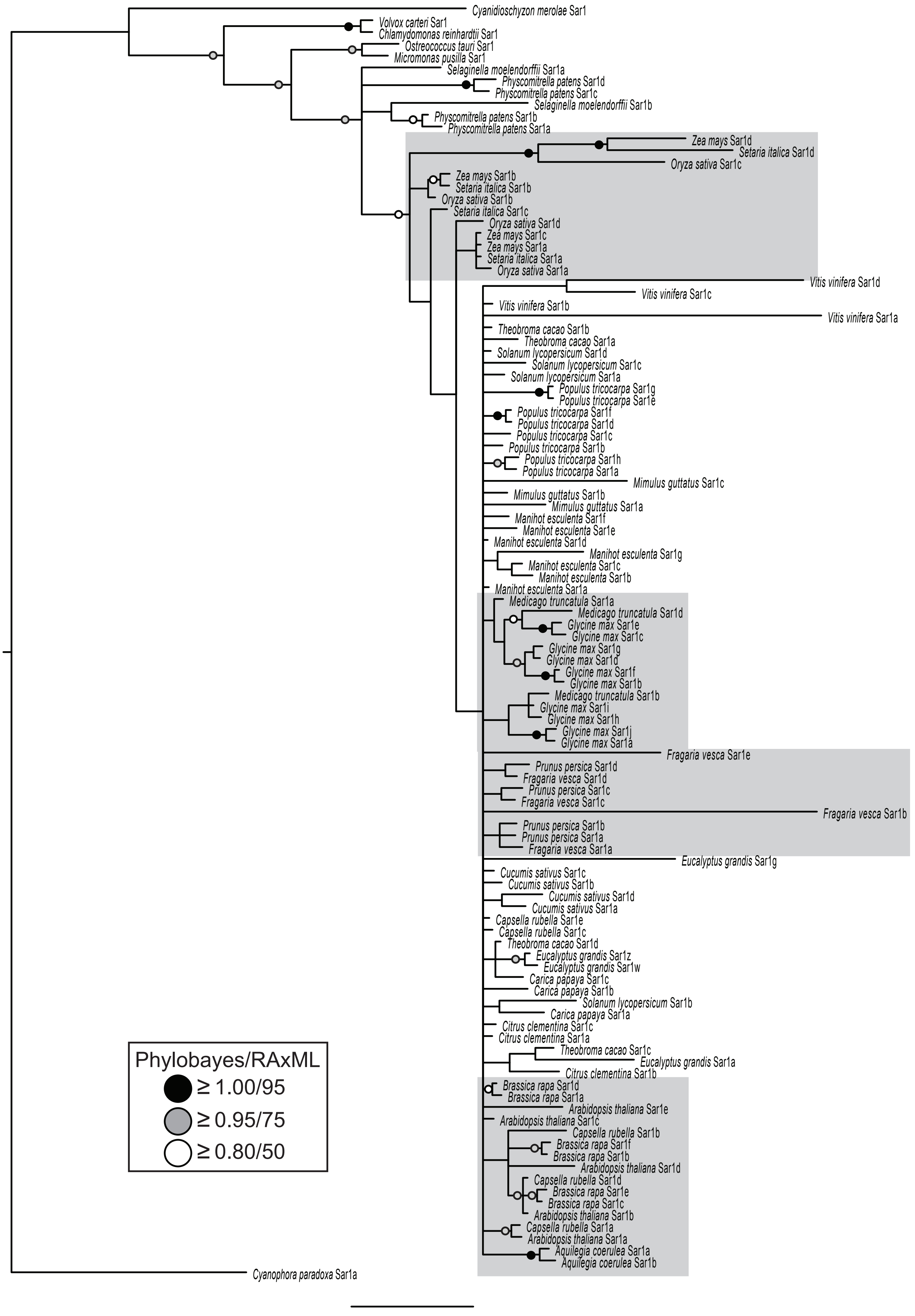

Schlacht and Dacks Figure S8

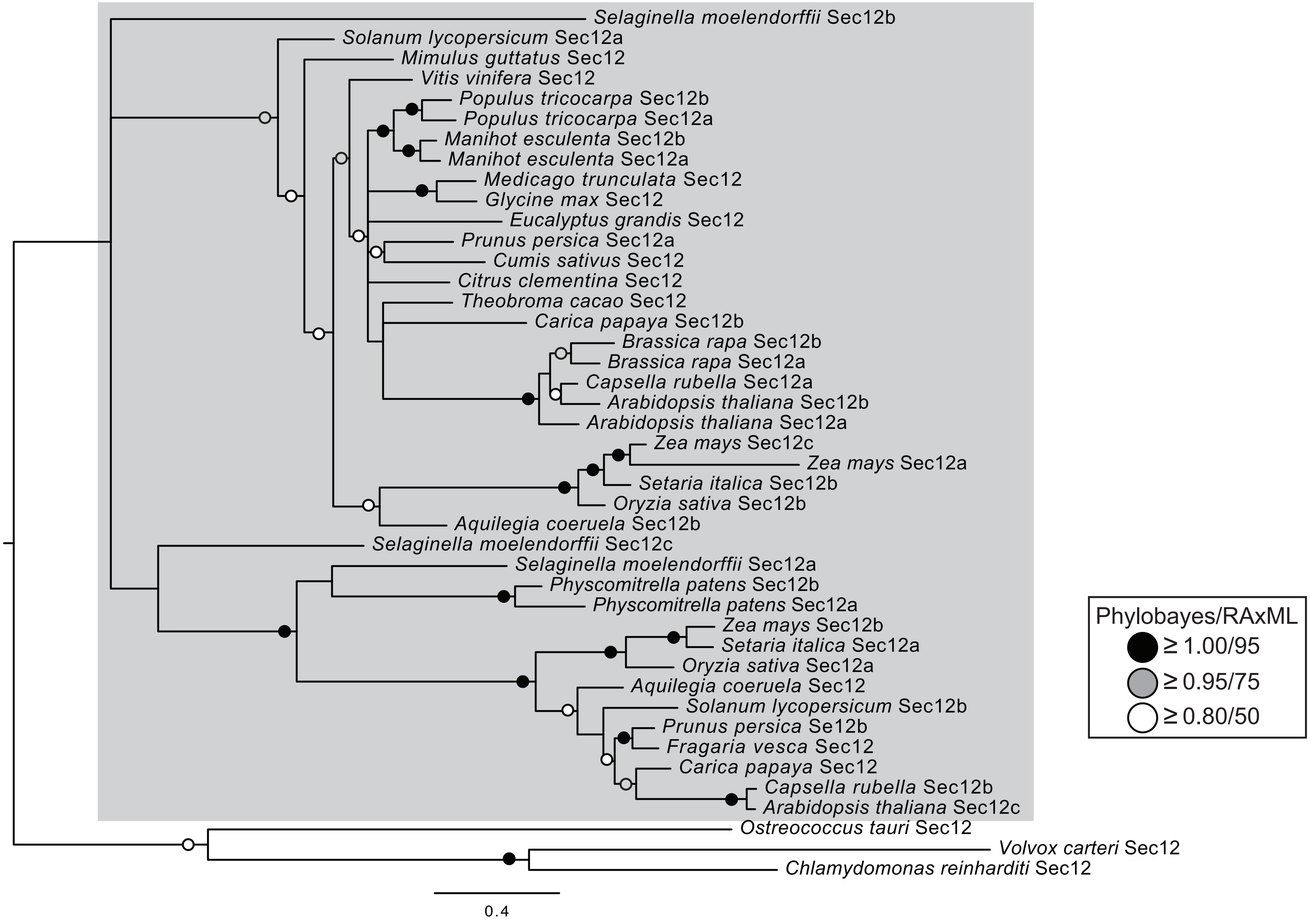

Schlacht and Dacks Figure S9

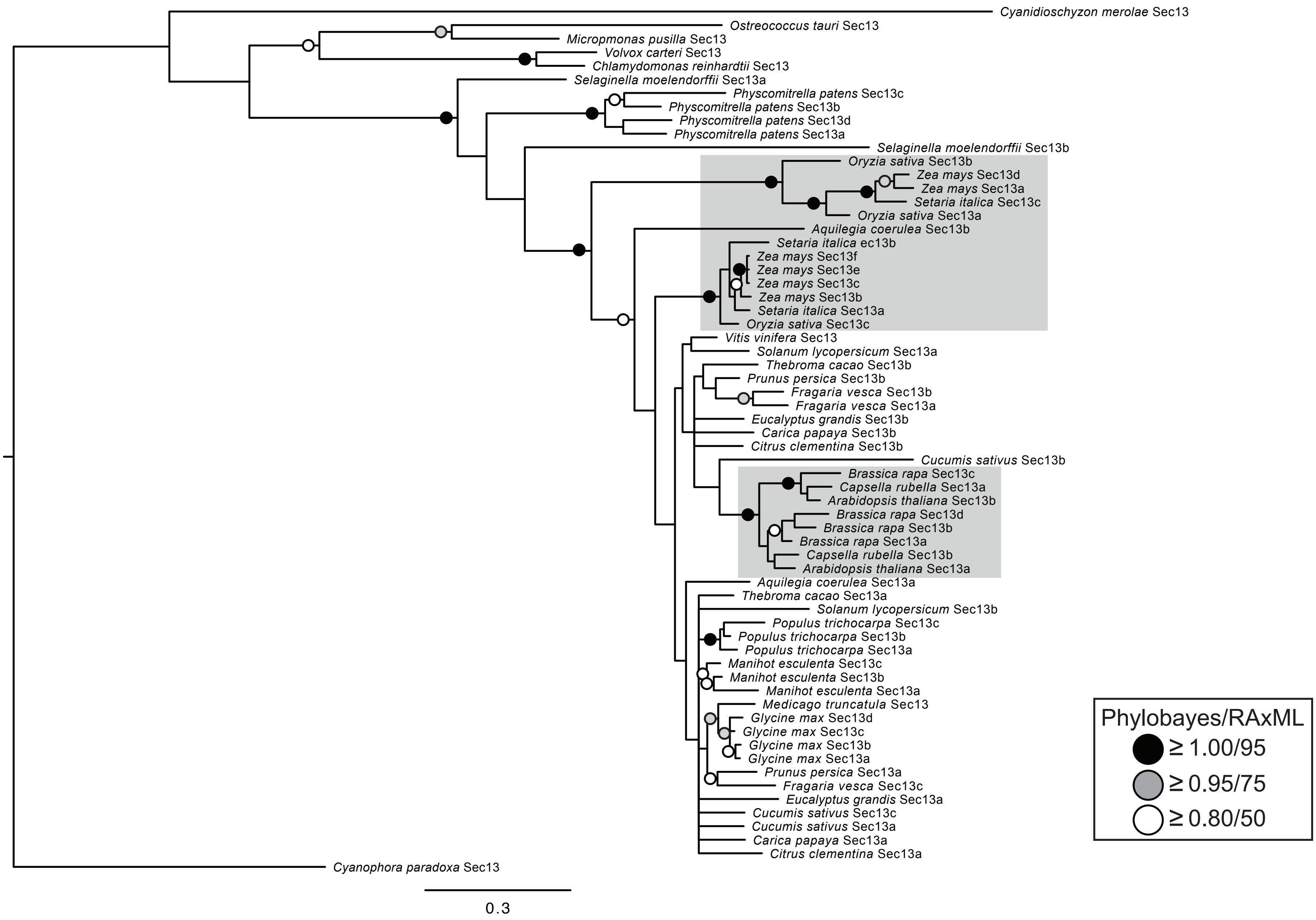

Schlacht and Dacks Figure S10

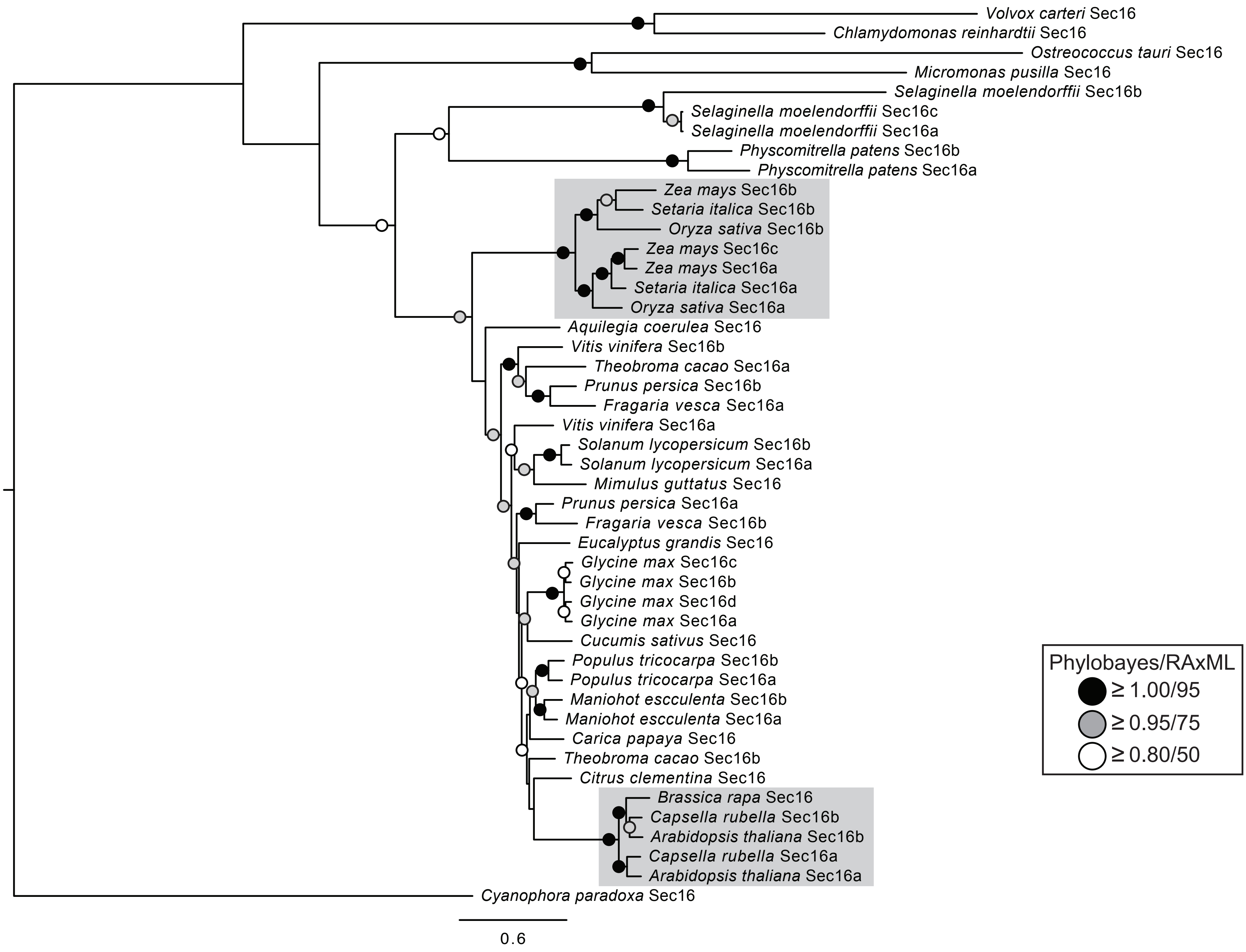

Schlacht and Dacks Figure S11

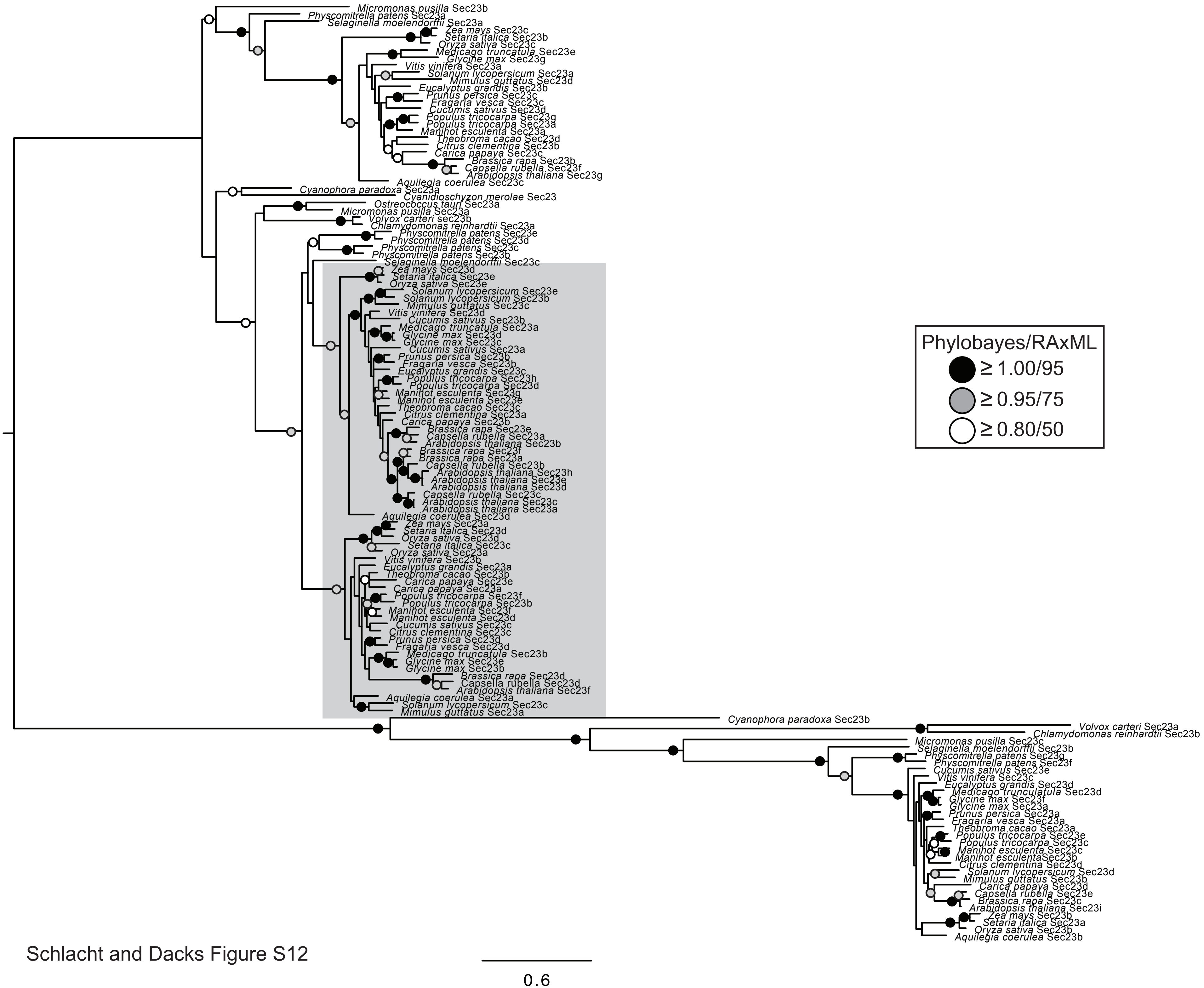

Schlacht and Dacks Figure S12

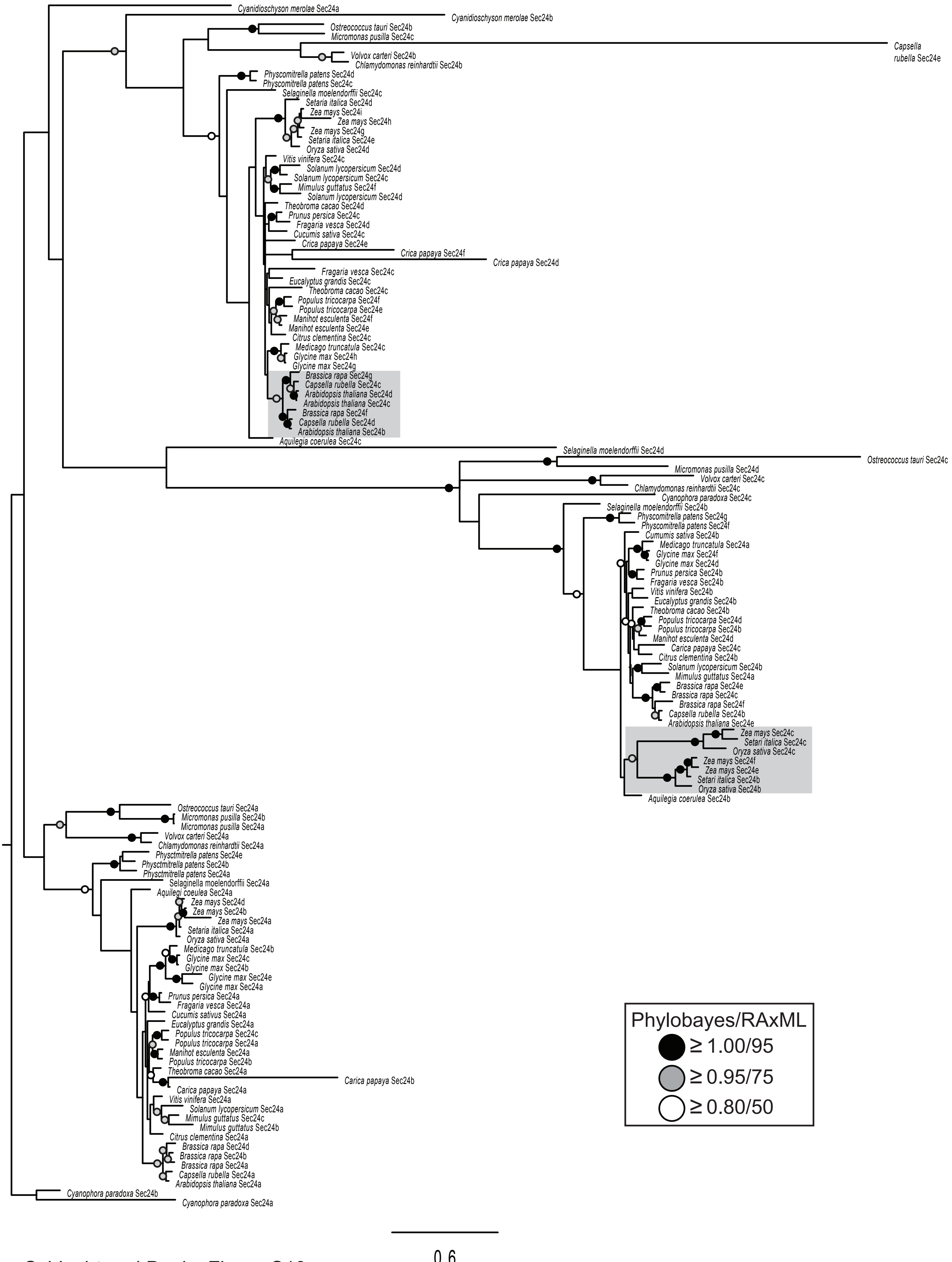

Schlacht and Dacks Figure S13

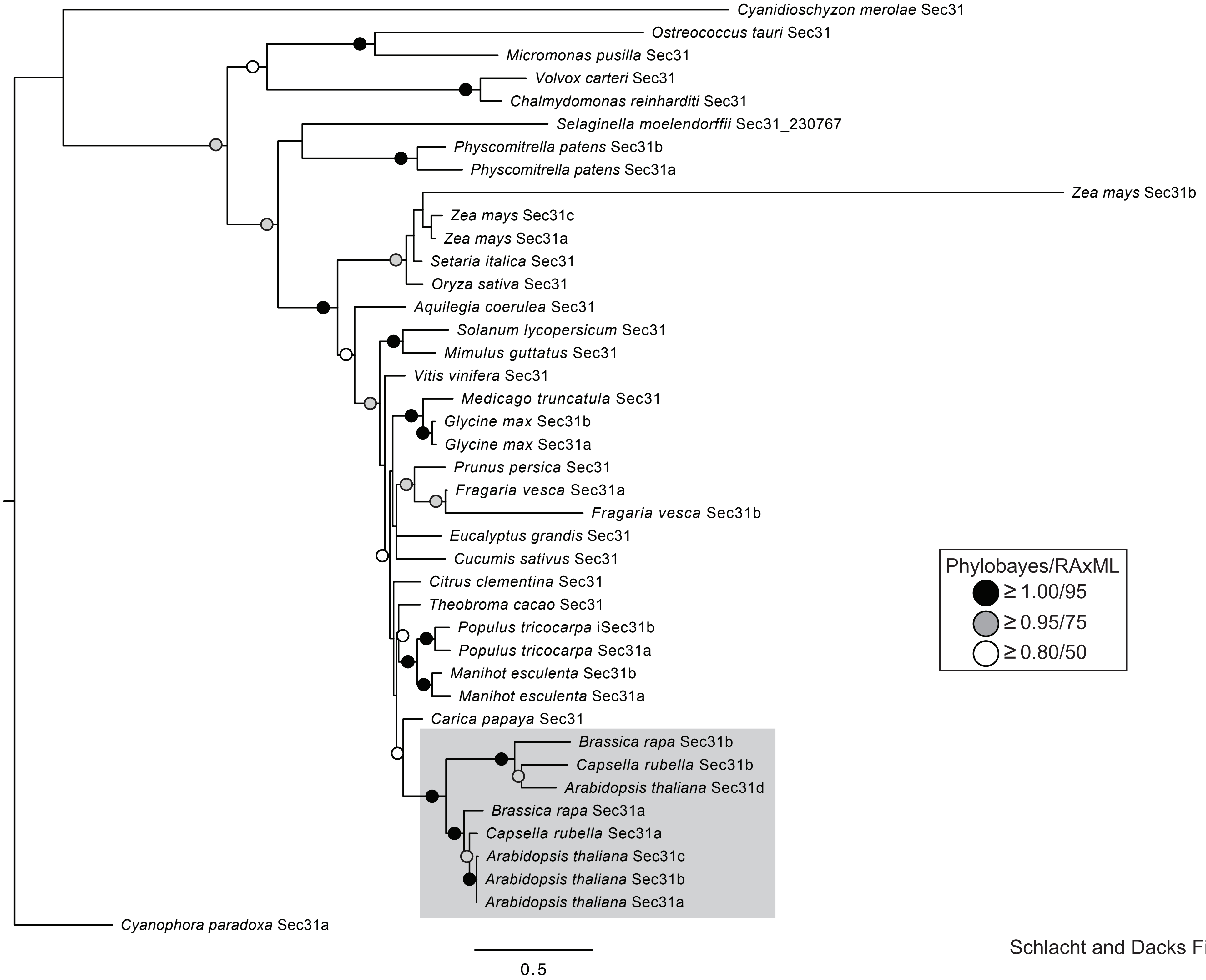

Schlacht and Dacks Figure S14

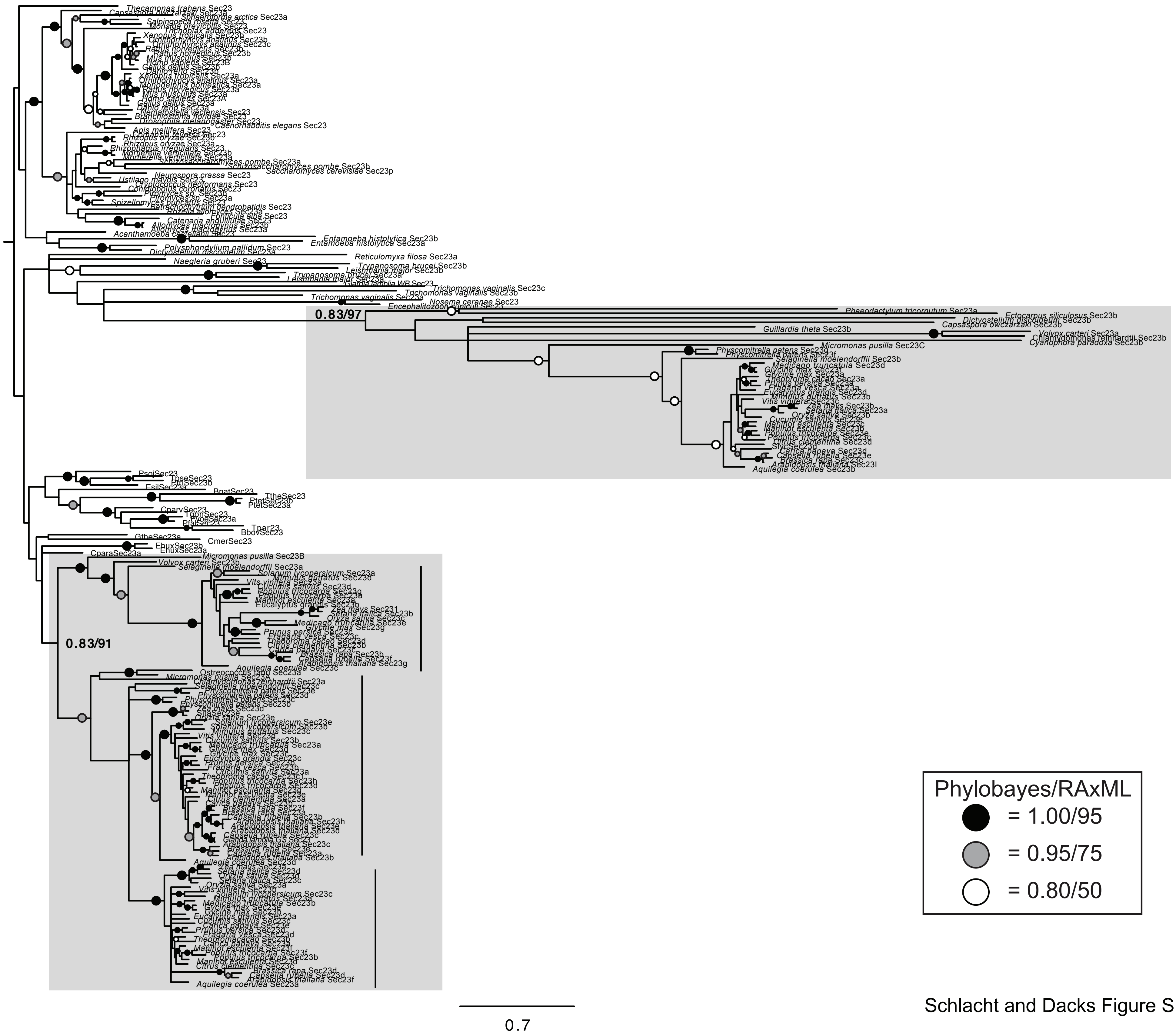

Schlacht and Dacks Figure S15

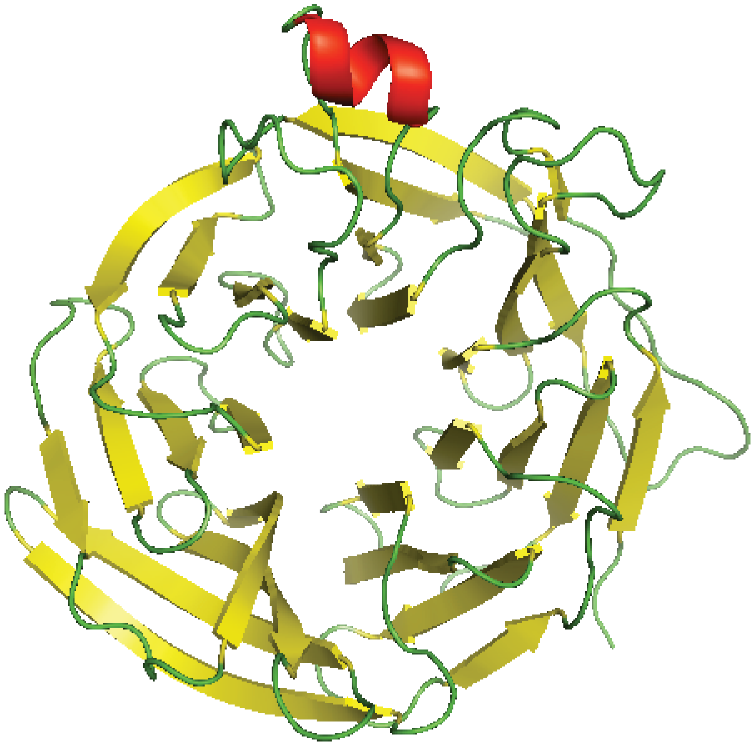

Schlacht and Dacks Figure S16

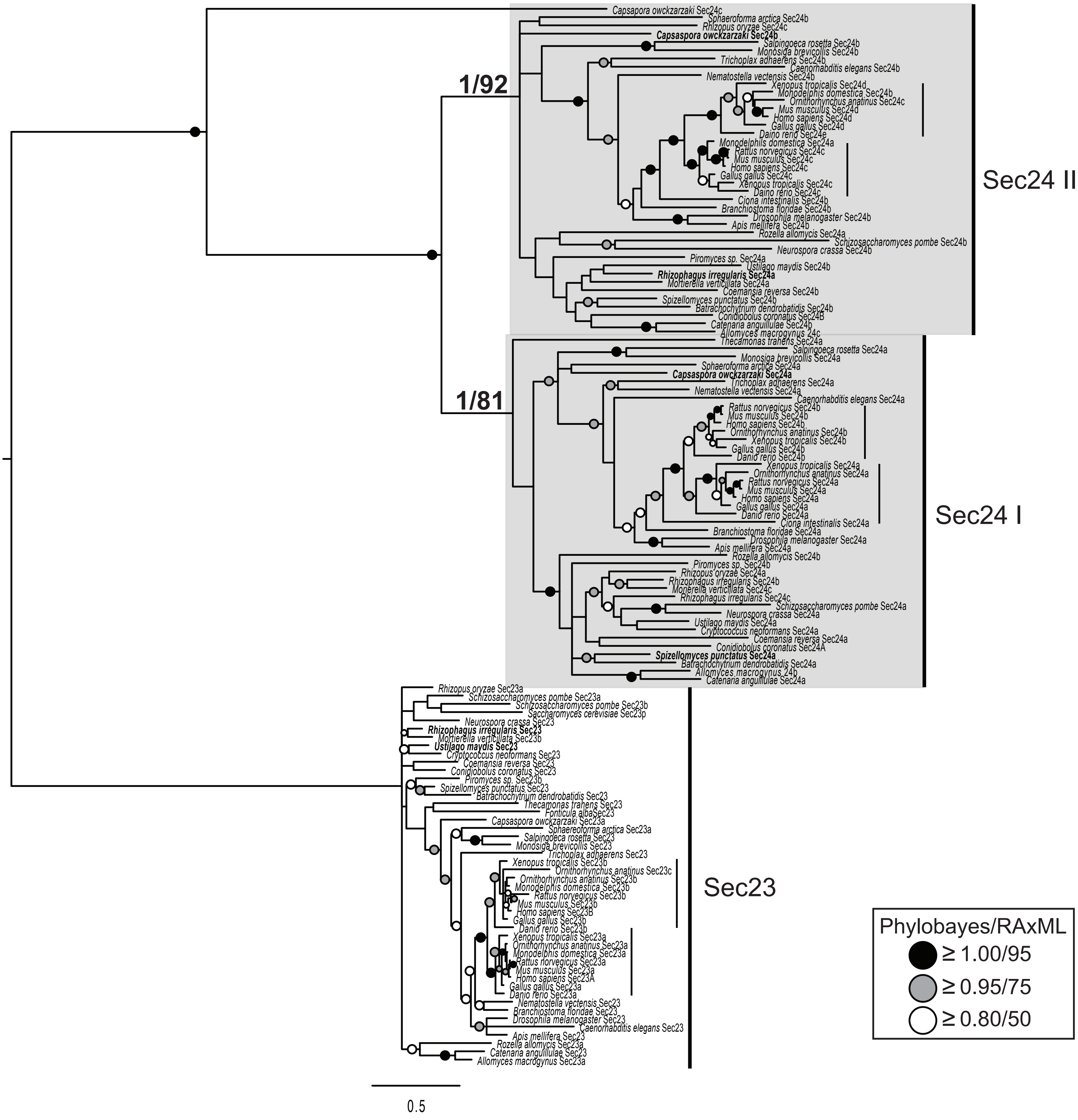

Schlacht and Dacks Figure S17

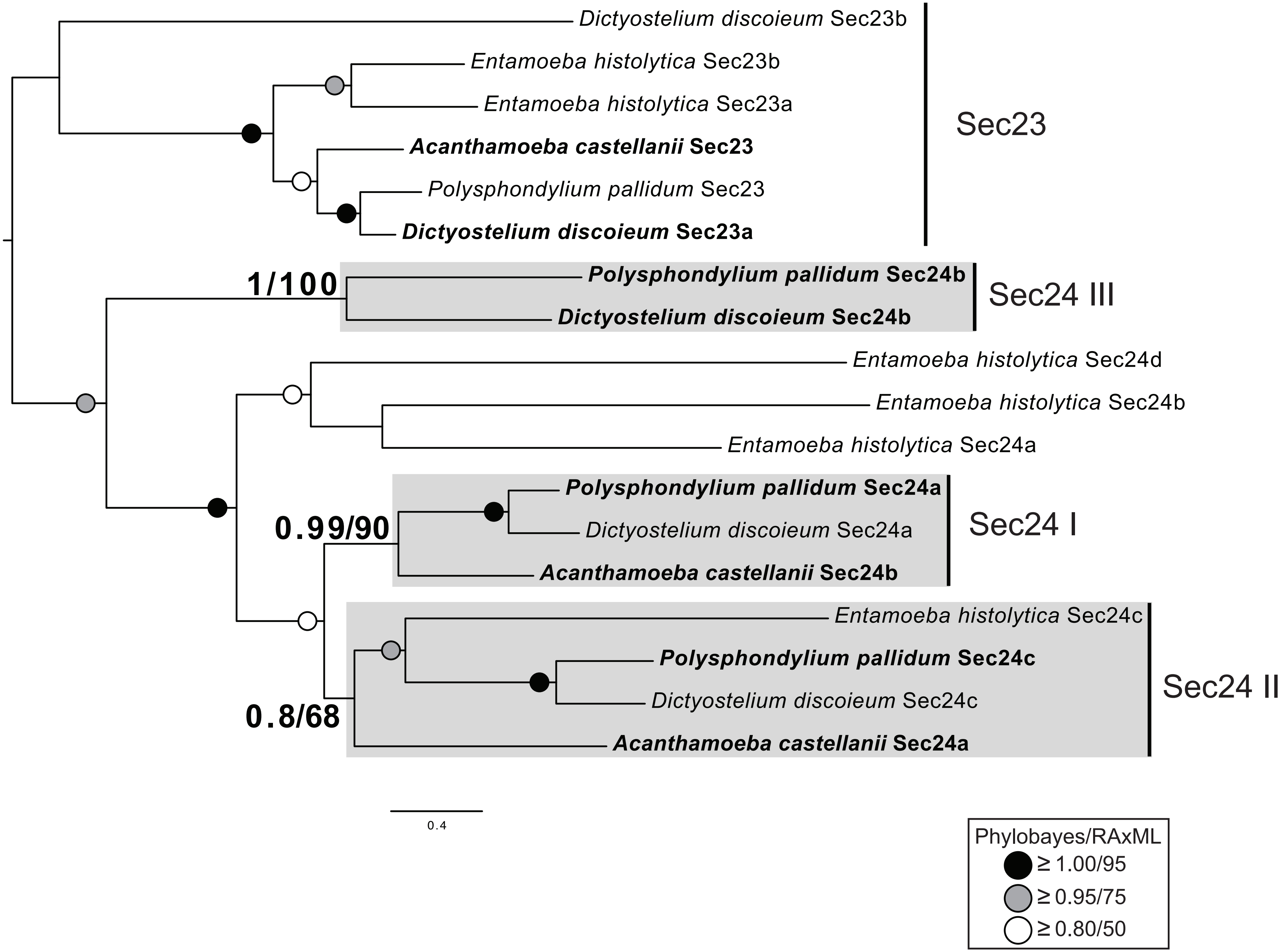

Schlacht and Dacks Figure S18

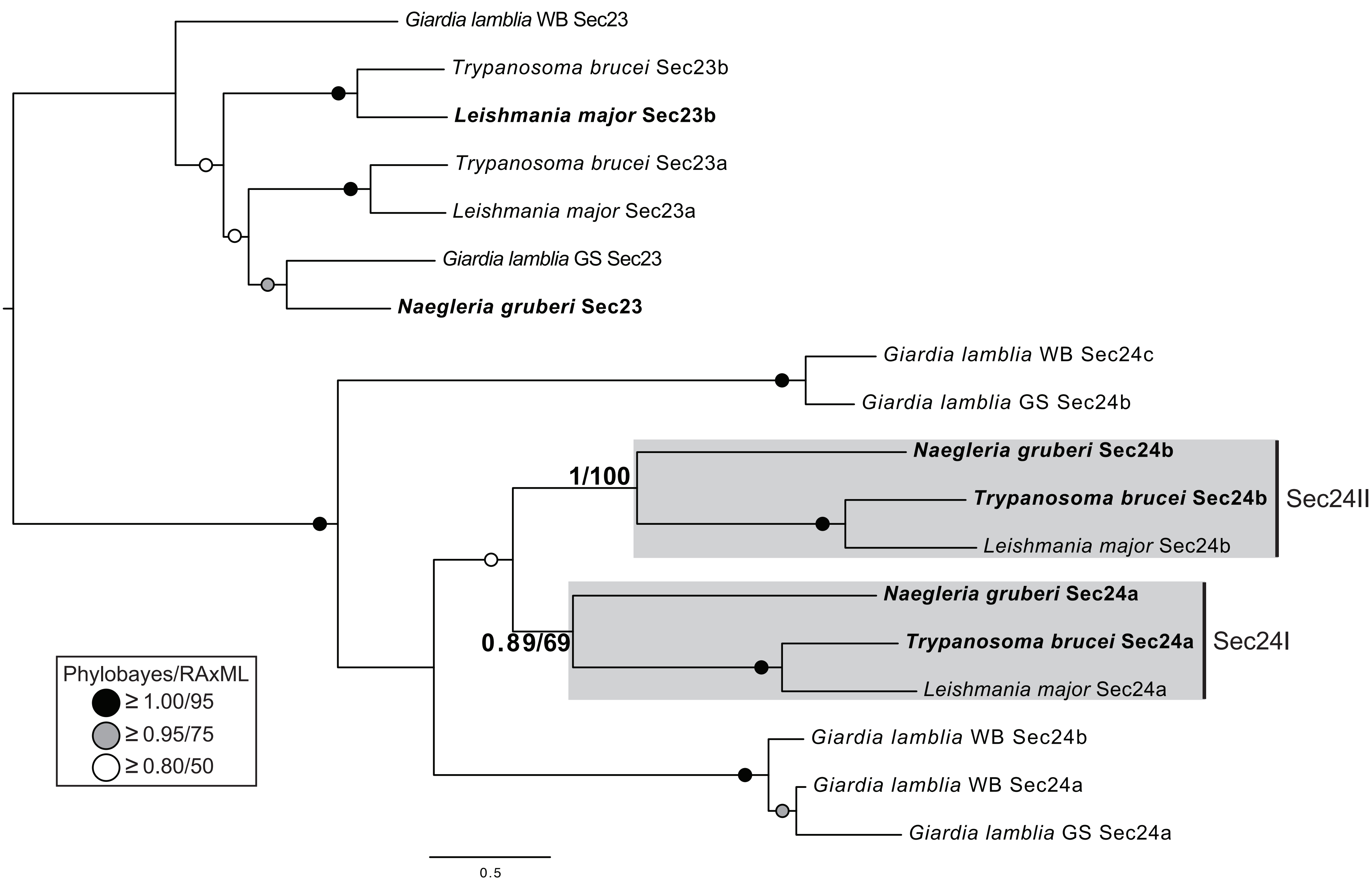

Schlacht and Dacks Figure S19

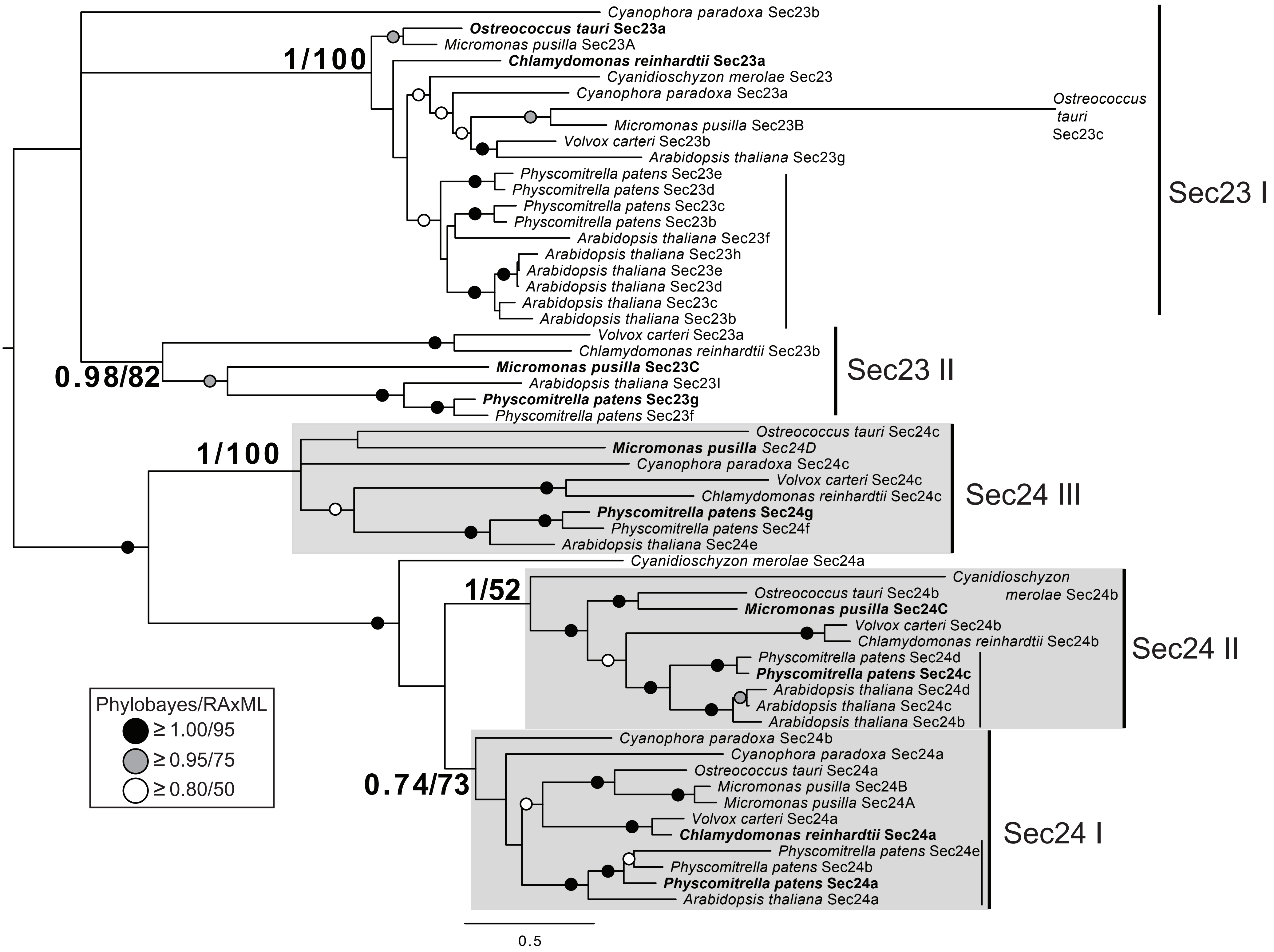

Schlacht and Dacks Figure S20

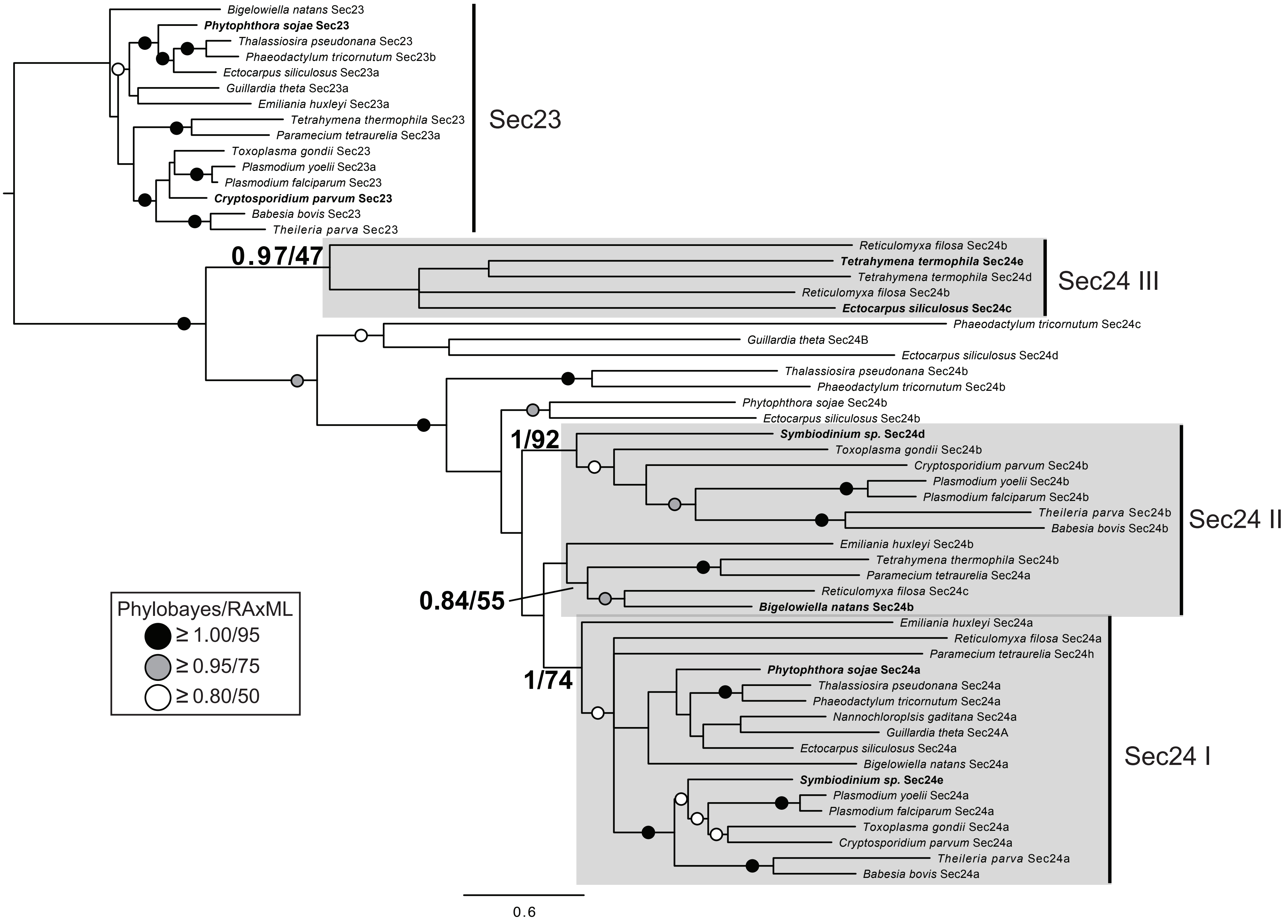

Schlacht and Dacks Figure S21

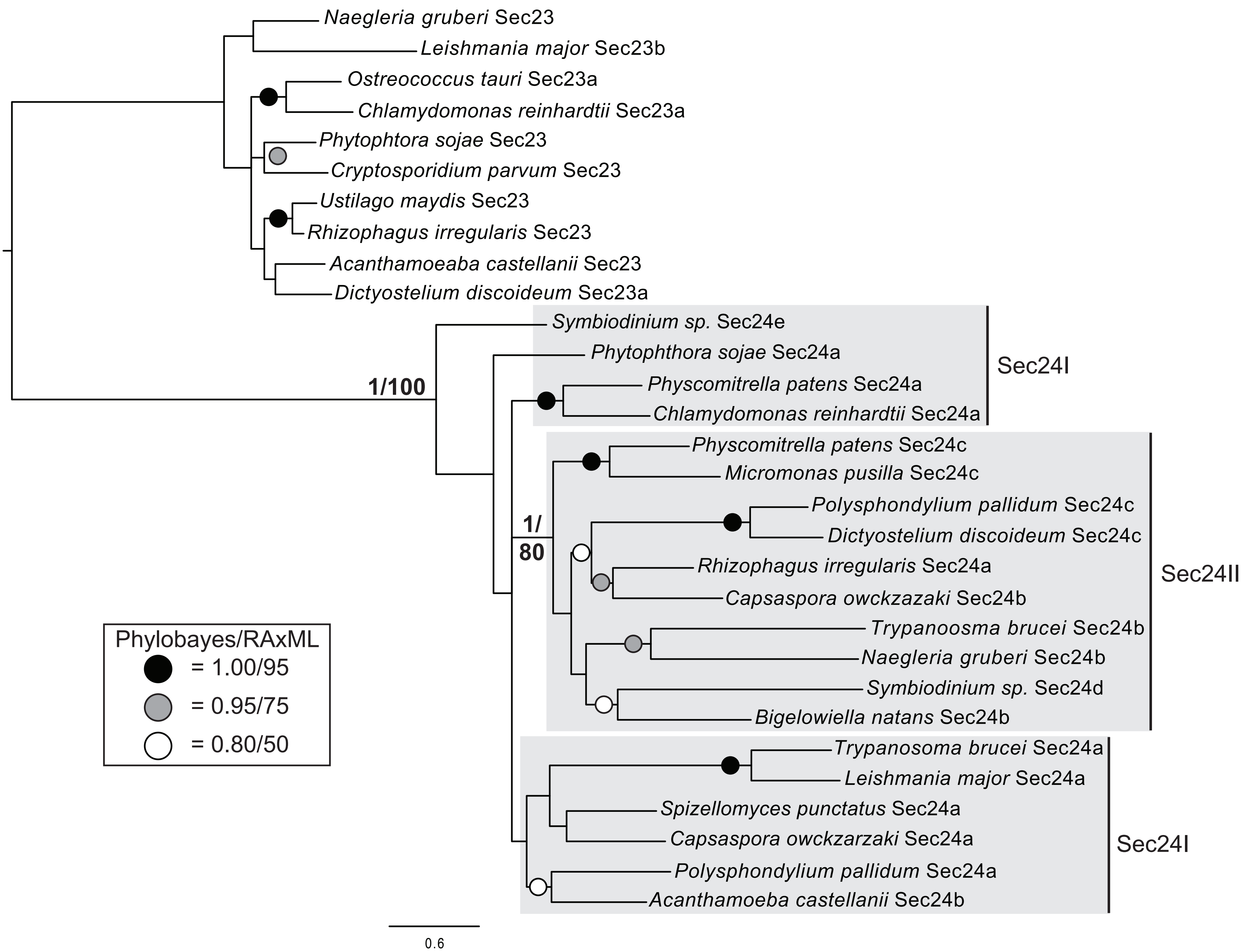

Schlacht and Dacks Figure S22

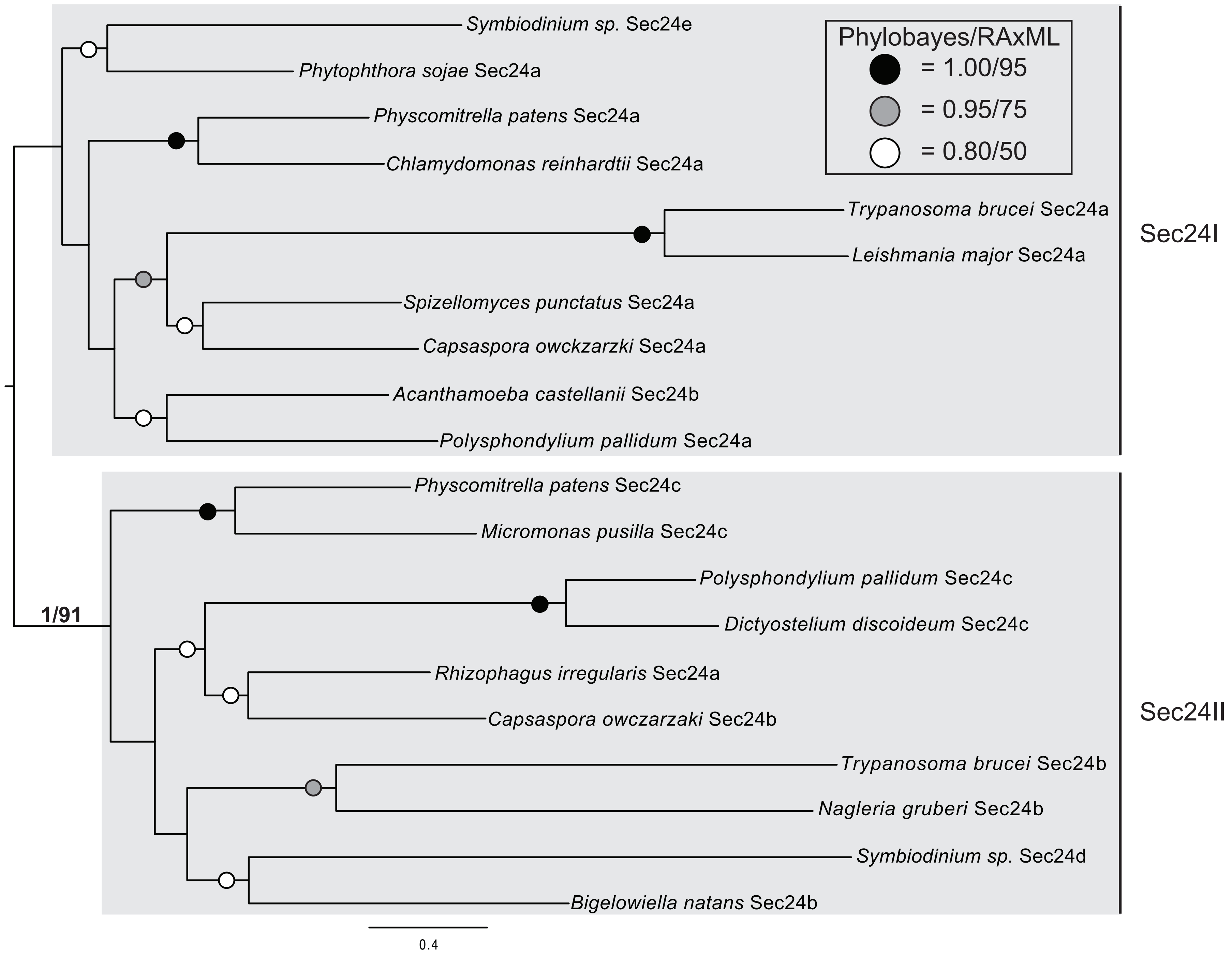

Schlacht and Dacks Figure S23

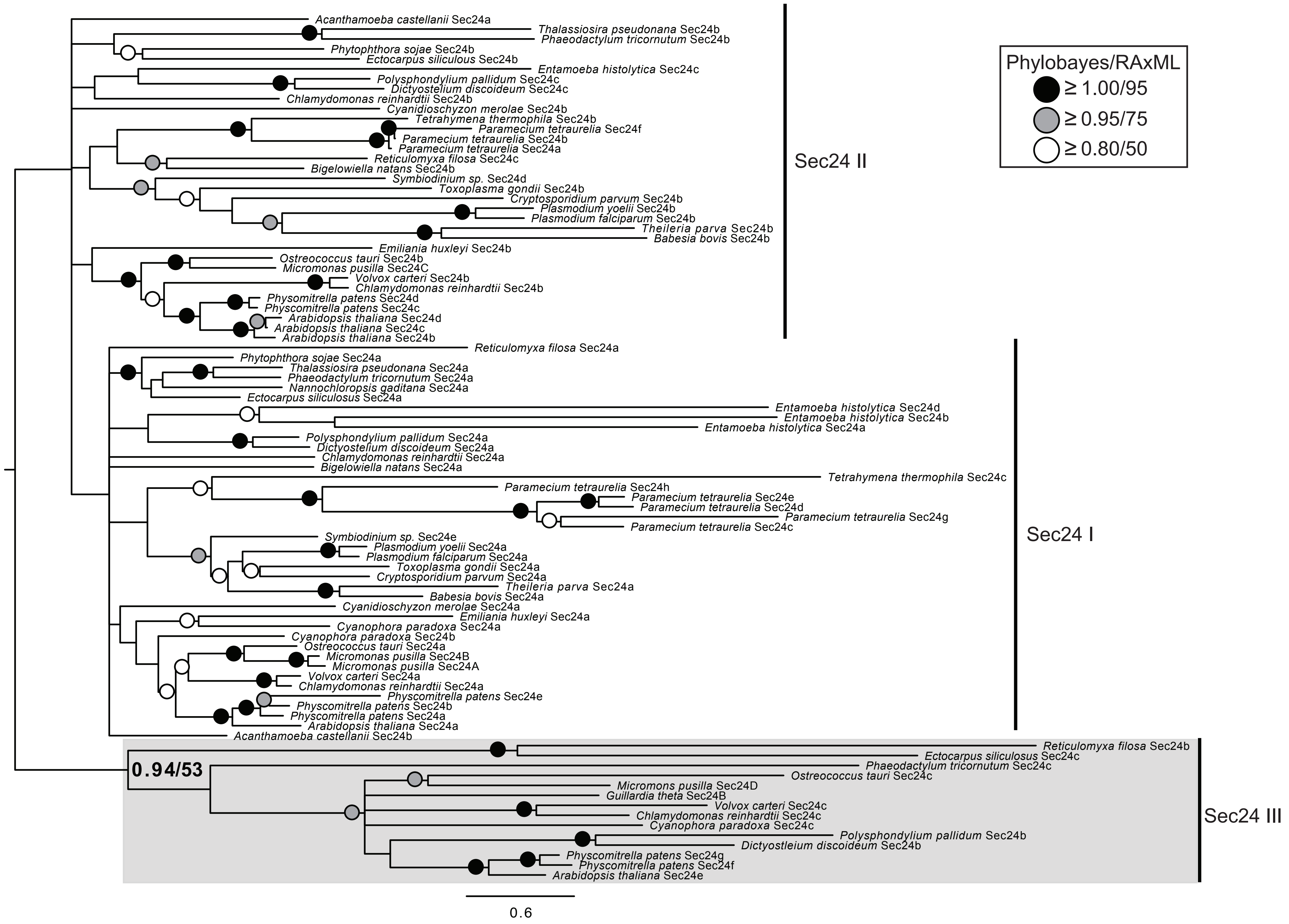

Schlacht and Dacks Figure S24
